# General Index to the British and Foreign Medical Review

**Published:** 1848

**Authors:** 


O'SHAUGHNESSY, Dr., on narcotine viii 263

surgery of the jaws . . xx 195

OSIANDER, his bags for prolapsus uteri iii 134

his history of midwifery . xiv 80

on signs of pregnancy . . iii 247

uterine tubercles . . xii 558

Os Incæ of Peruvian skulls . . xviii 384

Osmazome, in broths and soups . xvii 22

Os sacrum, exostosis of . . xi 501

Osseous deposits, in serous membranes iv 53

structure, inflammation of . xii 155

of fishes, remarks on . xxiii 491

substance, hypertrophy of . xvi 383

system, diseases of, in animals xxiv 87

tissue, distinct from cartilage . xxiv 389

first formation of . . xxiv 390

Mr. Tomes on . . xxiv 386

transformation, Dr. Gross on xxiii 68

Ossicles of the ear, ligaments of . viii 77

muscles of the . . viii 86

Ossicula of the ear, movements of xxii 287

Ossification, centres of, in animals . xxiii 478

intra-cartilaginous form of . xxiv 392

-membranous mode of . xxiv 390

of the iris, case of . . . xviii 421

encysted tumours . . xviii 369

the penis . . . . . xx 136

process of . . . . xxiv 390

report on the . . . xvii 263

Ossifications in the heart . . ii 326

Osteitis, condensing, nature of . ii 536

M. Richet on . . . . xxi 128

Prof. Gerdy on . . . . ii 536

ulcerating, or caries . . ii 536

with exostosis . . . . ii 537

with periosteitis . . ii 536

Osteoid tumour, description of . xix 538

tumours, Prof. Müller on . xix 537

Osteology, illustrations of . . vii 537

Osteoma frontalis, Dr. Huss on . xxii 462

Osteomyelosis, Prof. Albers on . xvi 382

Osteonabrosis, atrophy of bone . xii 159

Osteophysis, description of . . xiv 544

frequent in Austria . . xiv 544

Osteopsathyrosis, fragility of bone xii 159

Osteosclerosis and osteoporosis . xii 158

Ostrich, grunting sound of, cause of ii 600

Os uteri, on artificial dilatation of iv 260, iv 400

and vagina, laceration of . ii 90

cartilaginous, incision of . vi 235

closure of, case of . . . xiii 566

treatment of . . . xxi 328

dilatation of, Burns wrong on ii 84

Dr. Kilian on cutting open the iii 414

excision of the . . . . xvii 513

imperforate, cases of . . viii 55

incision of, in prolapsus . . vi 235

occlusion of . . . . xix 353

on dilating the . . . iii 413

pruritus in inflamed . . xvii 545

rigid, incision of . . . xxiii 425

use of the knife in . . xix 354

rigidity of, belladonna in . xiv 371

Os uteri, rigidity of, bloodletting in ii 79

state of, after labour . . iv 466

in pregnancy . . . iv 455

successful incision of . . xxiii 281

Otaphone, an instrument for the deaf vi 199

Otitis, catarrhal internal . . viii 92

chronic, remedy for . . xii 549

purulent internal . . . viii 92

Otitis externa . . . . . iii 89-90

interna . . . . . . viii 91

Otoconies . . . . . . viii 81

Otolites . . . . . . viii 81

Otoplastic, etymology of . . vii 411

operation . . . vii 411, xxi 302

Otter breed of sheep in America . vii 376

OTTLEY, Mr., his Life of John Hunter iv 75

on cysticercus . . . . xx 84

OTTO, Professor, on Copenhagen . ix 216

on cutaneous apoplexy . . viii 552

influenza in Copenhagen . v 558

medicine in Denmark . iii 565

rheumatism . . . . ii 252

state of medicine in Denmark ii 285

Ova, changes of cells round, post coitum xvi 521

changes of, in Fallopian tubes . xvi 529

detachment of, from the ovaries xvi 420, 556

discharge of, in menstruation xix 89

escape of, without coitus . xix 89

experiments on . . . xix 92

expulsion of, in menstruation . viii 530

first trace of villi in . . xvi 547

in relation to menstruation . xvii 271

limitation of, in human female x 73

maturation of, report on . . xxii 288

of man, &c., before fecundation xvi 513

periodical maturation of . . xix 84

processes following discharge of xxii 290

Oval envelopes, changes of, in the tubes xvi 529

Ovaria, diseases of, Dr. Meigs on . xxi 88

report on xvii 547, xx 545, xxiii 295

dropsical, on excision of . xvii 237

excision of, cases of . xvii 237, 240

reflections on . . xvii 238-40

results of . . . xvii 238

extirpation of . . . xvi 387

cases of . . . xvi 388-401

danger of . . . xvi 402

Dieffenbach's . . . xvi 399

Dr. Dohloff's . . . xvi 401

Dr. Chrysmar's . . xvi 396-98

Dr. Granville's . . xvi 396

Dr. Macdowell's . . xvi 394-96

Dr. Quittenbaum on . xvi 395

Dr. Ritter's . . . xvi 395

Dr. Rogers's . . . xvi 395

Dr. Smith's . . . xvi 394-95

in a negress . . . xvi 395

Martini's . . . xvi 400

Mr. Jeaffreson's . xvi 392, 401

M. L'Aumonier's . . xvi 393

Mr. Lizars' . xvi 394, 397, 399

Mr. Walne's . . xvi 391, 402

report on . . . . xx 546

Ovaria, extirpation of, statistics of . xvi 392

inflammation of . . . ii 526

relation of, to spinal cord . xii 68

Ovarian and uterine influence, on . xxiii 191

capsule . . . . . . ix 11

conception, observations on . xvi 521

cyst, diagnosis of . . . vii 466

fatal operation for . . xx 89

malignant, symptoms of . vii 465

paracentesis of . . vii 467

cysts, extirpation of, Mr. Key on xviii 340

Lisfranc on extirpating xviii 33

rupture of . . . xvii 547

disease, absence of symptoms in ii 513

curable stage of . . iii 166

Dr. Ashwell on . . iv 373

Dr. Barlow's cases of . iii 166

remarkable cases of . iii 167

remarks on . . . iii 165

dropsy, bruit de soufflet in . vi 97

diagnosis of . . . xvii 547

Dr. Ashwell on . . iv 373

extirpation in, Dr. Ashwell on xix 358

extraction of sac in . vi 97

Mr. Jeaffreson on . . iv 477

Mr. Lee on . . . xxiv 512

novel mode of curing . xvii 547

tapping in . . . iii 139

dangerous . xxiv 513

treated by compression . xxiii 296

inflammation, case of . . ii 528

diagnosis of . . . ii 527

symptoms of . . . ii 527

operation, Mr. Heath's fatal . xvii 240

ovule, discovery of . . ix 8

early, of the bird . . ix 5

envelope of mature . . ix 5

pregnancy, case of supposed . xxiii 278

tumour, removal of an . . iv 477

tumours, Dr. Barlow on . iii 165

Dr. Bright on . . vii 464

four forms of . . . vii 464

operations for . . xx 101

use of iodine in . . iv 373

Ovaries, after cessation of menses . xix 89

after impregnation . . viii 39

cancer of . . . . xxii 307

diagnosis of . . xxii 308

conveyance of semen to . . xvi 521

function of, in menstruation . x 592

in hernial tumour, case of . xxii 195

of pregnant women described . iv 461

tumours of, in labour . . xi 503

Ovariotomy, conclusions of Mr. Lee on xxiv 514

reprobation of . . . xxii 383

Ovaritis from uterine injections . xi 246

Ovarium, diseased, removal of . xx 90

Ovary, changes in, on conception . ix 15

of during menstruation xxi 89

contact of seminal fluid with . xvi 521

diseased, extirpation of . . xiii 547

muriate of lime in . . iii 139

percussion in . . . iii 139

Ovary, diseased, prognosis difficult in iii 139

various remedies for . iii 139

dropsical, removal of . . xxiii 297

eight days after conception . i 239

encysted, dropsy of . . vii 464

enlarged, leeches in rectum for xxiv 512

natural cure of . . iii 138

enlargement of . . . iii 165

case of . . . . iii 138

its obscurity . . . iii 138

Hamilton on enlargement of . iii 138

human, its consistence . . i 610

its relation to menstruation . x 70

of the bird . . . . ix 4

rarity of spermatozoa in . . xvi 520

stroma of . . . . ix 11

Over-eating, effects of . . . xi 232

Oviduct of birds, ciliary motions in i 510

Ovisac of Dr. Barry, or tunica propria xvi 527

of ovum, genesis of . . ix 11

Ovology, new observations on . viii 42

Ovum, albuminous substance of . ix 23

and embryo, physiology of . xix 587

as to the theory of cells . . xvi 519

changes in, post coitum . . xvi 522

correlation of changes of . xvi 541

development of . . . ix 1, 507

in uterus . . . xvi 542

J. Hunter on . . . xv 216

report on . . . xix 565

the brain in . . . ix 18

discus proligerus of . xvi 523, 525

diseased, causes abortion . vi 83

early development of . . v 555

external membrane of . . xvi 515

extract from Müller on the . ix 22

fecundation of . . xvi 520, 556

first formation of . . . xvi 519

rudiments of . . . ix 4

formation of the embryo in . ix 508

germinal spot of . . . xvi 517

vesicle of . . . xvi 517

gradual changes in . . ix 10

granules of . . . . ix 12

halving of, after impregnation xxiv 129

human, its situation and size . i 611

minute vesicle in the . i 611

in the ovary, anatomy of . i 610

its sameness in the vertebrata v 112

meeting of semen and . . xvii 272

nature of action of semen on . xvi 522

nuclei in cells of . . . xvi 525

of mammalia and birds . . ix 9

of the dog and rabbit . . xvi 549

of the mammifera, report on . xvi 513

on Dr. Barry's tunics of . xvi 514, 523

ovisac of the, genesis of . . ix 10

plate of developments of . ix 1, 31

primitive streak in the . . ix 18

process of cell-formation in . xvi 525

progress of, post coitum . . xvi 524

report on the . . . xvii 270

Schwann on the structure of . ix 508

Ovum, serous covering of the . . ix 20

vitellary membrane of . . xvi 515

yelk, and germ-vesicle of . ix 5

and its cells . . xvi 514, 534

zona pellucida of . . . . xvi 515

Outlines of human pathology, by Mr. Mayo iii 55

Owen, Prof., his great labours . xx 377

his Hunterian Catalogue . xv 195

lectures . . . . . xxiii 472

Notes on Hunter . . vii 523

Odontography x 208, xx 376, xxi 62

of the Hunterian museum . xv 195

on nervous action . . . v 538

the brain and spinal cord . v 537

lobster and pagurus . xv 212

structure of teeth . . vi 578

teeth . . . . . viii 158

Ox, bony process in its heart . i 435

heart of, weight and dimensions of i 435

varieties of, in different countries xxiv 60

Oxalate of lime, in the urine . . xx 92

pathology of . . xx 64

urinary deposits . xvi 316

Oxalic acid, action of . . . ii 284

formula for . . . xii 539

its effects as a poison . ii 284

morbid production of . xvi 484

old mortar an antidote for xviii 553

on poisoning by . vii 454, xx 325

secretion of, causes of . xx 67

test for . . . . xvi 78

use of . . . . . xii 539

Oxalic-acid diathesis, cause of . xi 342

diet in . . xi 342

treatment of . xv 511

symptoms of . xx 66

urine in . . xi 342

urine, characters of . . xx 65

Oxford, case of, Dr. Mayo on . xix 38

criminal case of . . . xvi 106

Oxford, Dr. Marx's visit to . . xv 26

University, education at . . x 542

Ox-gall, stomachic virtues of . . xvi 148

Oxide of bismuth in yellow fever . iii 217

Oxides, necessary to vegetation . xi 441

Oxygen, as an element of food . xvii 3

-gas, conducive to putrefaction ii 396

or vital air, remedial use of . xix 232

Oxymel, remark of Dr. Collier on . iv 115

Oxyprotein, coagulable lymph composed of . . . . . xviii 263

Oysters, difference of doctors on . xvii 47

Ozena, on the cure of . . . xii 549

treatment of, by Dr. Heron . i 270

Pacchionian bodies, observations on xxi 455
structure of . . xxiii 142
Pacinian corpuscles, electrical theory of xix 83
nerve fibres in . . xxii 274
of the nerves . . xix 78
remarks on . . xxi 456
Pads, hernial, on the proper form of xiv 102
Pads, hernial, triangular . . xiv 103
new, for radical cure of hernia iii 234
Padua, diseases at, in 1834-5 . x 206
medical statistics at . . xxiv 361
topography of . . . x 205
Pagan, Dr., his jurisprudence of insanity x 129
Paget, Mr., his Records of Harvey xxiii 249
his report on anatomy xix 249, 563, xxii 261
and physiology xvii 249, xxi 541
physiology . . xiv 259
on a seminal cyst . . . xx 100
blood-vessels in tendons . viii 594
bone . . . . . ix 272
malformation of the brain . xxiv 323
obstruction in pulmonary arteries xx 93
of arteries . xxiii 413
spots of the heart . . xi 448
symmetrical disease . . xv 149
the microscope . . xv 542
the relative size of arteries . xiv 574
the study of medicine . xxiii 242
Paine, Dr., false assertions of . xv 136
his contradictions . . . xi 387, 392
faults of reasoning, &c. xi 398
former praise of this Review xv 135
Materia Medica . . xiv 213
medical commentaries . xi 382
original views on life . . xv 144
personalities on this Review xv 134
review of Louis . . xi 401
symbolic formulæ . . xiv 214
on his doctrines . . . xi 383
on vitality . . . . xv 134
and vital forces . xi 385, 390
reluctant strictures on . . xiv 213
*versus* the microscope . . xi 384
*versus* Dr. Carpenter . . xi 387
Pain, and pleasure, feelings of, seat of xxii 512, 514
Unzer on . . xxiv 197
cause of intense, in cancer . iii 24
in animals, difference of . viii 265
Mr. Walker on the use of . xiii 208
nervous, of left side . . iv 132
production of, modes of . . xix 99
Pains, on inflammatory and neuralgic iii 25
undefinable and intractable . iv 132
Painters, paralysis of . . . xvi 256
Painter's colic, cause of atrophy in ii 463
M. Chevallier on . ii 573
Paintings, fine, before pregnant women ii 367
Palæontology, mammalian, remarks on xxi 478
Palate, cleft, case of operation for . ii 498
cause of deafness in . viii 96
knot-maker for . . ii 496
Mr. Fergusson's paper on xix 415
new forceps for . . ii 495
new instruments for . ii 495-6
observations on . xix 408, 415
operation for . . . ii 493
origin of . . . . xx 134
plastic operations on . xix 412
suture, instrument for . ii 496

Palate, cleft, what prevent operating for ii 493

fissures of, leaden wire for . xxi 307

operation for . . . xi 196

hard, on the nerves of the . xiv 222

holes and openings in, treatment of xxi 306-7

nerves of the . . . xix 269

experiments on . xix 583

new muscle of nares and . xxi 558

soft, restoration of . . vii 411

suture, Dr. N. R. Smith on . i 265

needle for, *plate of* . i 266

Palatine fissure, Dieffenbach on . xxi 307

Palermo, statistics of insanity at . vii 1, 51

PALEY, Dr., on mortification . ix 571

Pali, and real plague, compared . ix 234

plague, description of . . ix 234

endemial causes of . . ix 235

origin of . . . . ix 235, 236

Palmar arteries, wounds of . . iv 157

contraction, case of . . iv 172

cord, division of, by Dupuytren iv 169, 172

hemorrhage, case of . . v 463

on arresting . . . v 463, 464

PALMER, Dr., his Dictionary of Medical Terms . . . ii 508, xx 207

PALMER, Mr., his edition of Hunter's works . . . iii 486, iv 75

his lectures of John Hunter . v 46

PALMIERI, ADONE, Eclectic of . i 546

Palpitation, an occasional cause of . vii 128

of heart, musk in . . . iv 399

use of creosote in . . . ii 169

Palpitations, chlorotic, diagnosis of . xiii 333

chlorotic, or anæmic . . ii 336-7

of heart, diagnosis of . . ii 337

inorganic . . . viii 465

of the studious and dissipated . ii 336

organic, diagnosis of . . xiii 333

prominence of precordia by . i 448

rheumatic and nervous . . ii 337

Palsied arms, condition of nerves in v 501

Palsy, cautions on bleeding in . . viii 490

danger of stimulants in . . i 27

Dr. Holland on treatment of . viii 490

Mr. Mayo on the nature of . v 107-9

simulation of, by soldiers . . xviii 121

Panaceas, cause of the favour of . xvii 472

PANCK, Dr., on cod-liver oil . . xiv 441

PANCOAST, Dr., his operative surgery xx 39

Pancreas, and its fluid, properties of xxi 563

and stomach, diagnosis of diseases of xviii 398

anormal hardness of . . . xviii 403

atrophy of . . . . . xviii 399

calculi in . . . . . xviii 399

cancer of . . . . . xxii 299

case of disease of . . . xv 151

diseases of . . . . . xx 244

diagnosis of . . . xviii 396

etiology of . . . xviii 389

hunger and thirst in . xviii 392

nervous affections in . xviii 394

prognosis of . . . xviii 399

sensations in . . . xviii 390

Pancreas, diseases of, statistics of . xviii 389

symptomatology of . . xviii 389

symptoms in . . . xviii 390-4

treatment of . . . xviii 399

vomiting in . . . xviii 392-3

wasting in . . . xviii 394

watery discharge from mouth in xviii 391

fatty degeneration of . . xviii 399

hyperemia of . . . . xviii 400

ileus from hypertrophy of . xii 538

inflammations of . . v 567, xviii 400

morbid softness of . . . xviii 403

pseudo-morphous diseases of . xviii 403

report on the . . . xix 277

scirrhus of . . . . xviii 403

simple hypertrophy of . . xviii 399

suppuration in . . . xviii 403

treatise on diseases of . . xviii 388

weight and dimensions of . iii 199

Pancreatic disease, with fever . . xviii 396

fluid, of an elephant . xxi 561

juice, digestive power of . . xxiii 392

with gastric disease . . xviii 395

hepatic disease . . xviii 395

nervous diseases . . xviii 396

Pancreatitis, acute, history of . . xviii 400

and gastritis, contrasted . . xviii 401

chronic and acute, case of . xviii 400

and scirrhus contrasted . xviii 404

symptoms of . . . xviii 402

PANIZZA, confirmation of his views . xiv 545

Pannus of the eye, treatment of . x 36

Panorama, medical . . . xxiii 86

PAOLI on watery vapour from the lungs i 241

Papel negroes of Africa . . . xxiv 447

Papers, medical, in British journals . xi 534

Papillæ of the tongue, as organs of taste ii 433

skin, described . . ii 432

functions of . . ii 435

PAPIN, and his digester, account of xiii 494

PAPPENHEIM, M., on ciliary motion xi 521

Papua, the organized productions of iii 370

Papuas, mop-headed, a pure race . xxiv 470

Papulæ, remarks on . . . ii 154

syphilitic . . . . xxiii 360

the order of . . . . xv 391

PARACELSUS, and his physiology . i 383

compound names of . . xiv 148

erroneous opinions on . . xiv 148

estimate of his character . . xiv 153

false accusations against . xiv 151, 153

general war against . . xiv 150

his defence of his style . . xiv 151

knowledge of drugs . . xiv 157

mode of prescribing . . xiv 152

notions of pathology . . xiv 155

opinions on medical men . xiv 153

tartaric diseases . . xiv 158

therapeutics . . . xiv 157

travels . . . . xiv 152

honour and gratitude due to . xiv 158

medical axioms of . . . xiv 156

career of . . . xiv 152

Paracelsus, medicine in the time of . xiv 154

on national medicine . . xiv 155

on the powers of nature . . xiv 156

real merits of . . . . xiv 147

summary of his exertions . xiv 153

the constant aim of . . xiv 158

translations of his works . . xiv 150

Paracentesis abdominis, history of . xvi 55

Paracentesis, in empyema, debate on xiii 369

opinions on . xx 450

of abdomen . . . . . xiv 254

of the brain, case of . . iii 311

of thorax, situation for . . xiii 370

on pneumothorax from . . xiii 371

Reybard's canula for . . xiii 371

thoracis, best spot for . . xviii 343

Dr. Hughes on . . xxiii 419

history of . . . xvi 53

Mr. Liston on . . . v 468

new trocar for . . xviii 344

remarks on . . . iii 71

success of . . . xviii 343

Paradox, an auscultic, Dr. Hudson on i 488

Parafibrine and bradyfibrine . . xxi 543

Paralysed limbs, contractile power of iv 422

parts, temperature of . . iv 425

Paralysis agitans, of children . . xx 556

Paralysis, and spasm, on transient . xxii 406

atrophy in . . . . . ii 463

case of, cured by trephining . xv 175

cured by galvanism . . xi 524

curious case of yawning in . iii 38

discussion on the nature of . v 107

Dr. Abercrombie on . . iii 320

Dr. Webster's case of . . xviii 364

effect of civilization on . . xviii 250

electricity in . . . xii 345, xxiii 405

electro-galvanism in . . xx 260

facial, agheustia in . . . xxii 282

case of, by Dr. Watson . xvii 133

cases of . . . . . vii 247

from tubercles in brain . vi 200

observations on . . xvii 133

use of emetics in . . vii 247

friction and exercise in . . iii 323

from cerebral hemorrhage . i 142

cold, Dr. Huss on . . xxii 468

lead, account of . . x 449-51

treatment of . . ix 435

mercury, remarks on . x 449

metastasis . . . xiv 340

peripheral nerves . . xvi 255

tubercles in the spine . i 576

visceral disease . . xiii 562

general, of the insane . vii 14, xx 29

hysterical, case of . . . iii 143

in children, report on . . xvii 555

in insanity, treatment of . . xx 30

interesting case of . . . xvii 266

involuntary movements in . ix 437

irritability in . . . . ix 438

observations on . . . xiv 340

of bladder, cantharides in . ii 549

Paralysis of bladder, cold water in . i 497

M. Civiale's practice in . i 497

treatment of . . viii 280

of face, myotomy in . . xiii 241

facial nerves . . . xiii 235

muscles of inspiration . . v 133

portio dura, case of . . xiii 553

seat of the placenta . . xv 105

sensation, case of . . i 261

sensation only . . . xviii 365

sensitive nerves, pain in . iii 22

serratus magnus, case of . xvii 99

on no morbid traces after . iii 328

partial, case of . . . vii 216

of the face, case of . . v 281

pain in, accounted for . . iii 21

prolonged emotion in . . iii 320

remarkable phenomena in . v 516

remarks on recovery from . iii 322

remediable cases of . . iii 321

sudden recoveries from . . iii 322

tincture of sebadilla in . . ii 499

torpor of thoughts in . . iii 320

treatment of . . . . viii 490

use of stimulants in . . iii 323

thermal baths in . . xiv 339

Paralytic cases, division of . . iii 320

limb, inflammation from heat in iii 15

lunatics, remarks on . . xix 288

Paraphymosis, reduction of . . xiii 565

use of belladonna in . . xii 556

Paraplegia, a peculiar cause of . xxiv 42

case of . . . . . . vi 261

Dr. Graves on . . . xvi 254

from peripheral nerves . . xvi 255

Mr. Key on . . . . vii 446

treatment of . . . . xx 260

use of setons in . . . xiii 261

Parasite mass removed from a child xii 376

of the skin, Mr. Wilson on . xxi 200

Parasites, frequency of, in man . ix 442

monsters so called . . . viii 30

the aphyllous . . . iv 19

the chlorophyllous . . . iv 18

vegetable, in the body . . xiii 485

within vegetables . . . iv 19

Parasitic animals, destruction of . xxiv 136

in man . . . xx 230

Professor Vogel on . xxii 336

formations . . . . xiii 232

on the diaphragm . xxiv 179

growths in India . . . xii 424

monster, curious contents of . xiv 64

origin of diseases . . . xx 406

plants, nutrition of . . iv 18

true and false . . . iv 18

Parasitoid formations, nature of . xxiv 135

Parchment-like mark in strangulation ii 420

Pareira brava, a lithontriptic . . ii 515

a powerful diuretic . . ii 515

discordant opinions on . ii 515

disparaged by Mérat . ii 516

M. Duparcque on . . ii 515

Pareira brava, mode of exhibiting . ii 515
ovarian disease cured by . ii 515
root, Sir B. Brodie on . . iv 106
Parenchyma of viscera, their structure i 395
Parenchymatous organs, functions of xvi 151
Parental habits, effect of, on progeny vii 378, 383
Parent Duchatelet, biography of iv 63-4
on prostitution . iv 63
Parents and offspring . . . vii 375
influence of, on the germ . xvii 247
Parietal bone, fracture of, in labour xxiii 300
Parise, M., his pathology of rheumatism i 254
Pariset, M., on improving constitutions v 406
Paris, Dr., his Pharmocologia xvi 188, 205
inconsistencies of . . . v 400
on Diet . . . . . v 380, 398
unjust omissions of . . v 399
Paris, account of the asylums of xix 281, 594
bastardy and mortality in . xiv 455
English students at, book for . i 501
excessive use of leeches in . iii 389
graphic account of cholera in . i 213
hospices for the old in . . i 281
private lunatic asylums of . xix 608
prostitution in . . . . iv 63
Royal Academy of, memoirs of xxi 408
Schlegel's description of . v 371
Society, Memoirs of . . vi 28
Parisian fever, inferences respecting xii 304
hospitals, account of . . i 281
medical degree, studies for . i 494
report on gelatine as food . xiii 486
surgery, Dr. Markam on . xi 218
Parke, Judge, his decision on life, &c. iv 93, 95
Parker, Mr. Langston, on syphilis xxi 495
on syphilitic diseases ix 239, x 381, 419
the stomach . . vii 115, 117
ulcer of the stomach . . vii 244
Parkes, Dr., on dysentery and hepatitis . . . . . xxiii 146
Parkin, Dr., on epidemic diseases xiii 497
Parkin, Mr., information on ague to v 560
on epidemic cholera, &c. . iii 178
on gout . . . . . xii 507
strictures on . . xii 507
Parliament, Houses of, ventilation of xix 18
Parnell, Mr., on chemical analysis xv 531
Parotid gland, case of extirpation of v 422
hypertrophy of . . xiii 567
inflammation of, in fever . vi 138
Parotitis, account of an epidemic . v 567
Paroxysmal fever in India . . xxiii 113
treatment of xxiii 117
Parr, Dr., his Medical Dictionary i 177
Parrish, Dr., his error on femoral hernia . . . . . iii 356
on strangulated hernia . . iii 355
Parson, American, anecdote of an . iv 57
Parturients, objection to the term . xvii 243
Parturition, accidental death of child in vii 139
aqueous discharge after . . xix 356
at tenth menstrual period . ii 404
case of unconscious . . v 255
Parturition, in privies, remarks on . vii 138
mechanism of . . . xii 462
peculiarities of . . . xix 65
period of . . . . . xxii 291
uterine vessels after . . vi 90
unconsciousness in . . vii 138
Partus siccus, case of . . . xvii 538
Par vagum, asthma in relation to . iii 28
barking from pressure of xiv 133
cough from disorder of . iii 27, 28
dyspepsia from disease of iii 28
dyspnœa from affection of iii 27
effects of division of . iii 28-29
functions of . . . xi 291
branches of . viii 265
in relation to the heart . xi 292
nerve, Dr. Ley on . . iii 27
observations on . . iii 220
of an ass, experiment on . iii 21
remarks on its functions . v 105
the nerve of hunger . xi 293
Pasquier, Dr., on a lunatic asylum ix 144, 178
Passavant, Dr., on vital magnetism xix 428
Passion, crimes from, punishable xxiv 240
Passions, anatomical expression of xviii 449-50
and desires, remarks on . . xxiv 238
and emotions, Unzer on . . xxiv 200
effect of, on health . . . xv 417
Patella, compound fracture of, case of ii 199
fractured, osseous reunion of . xiv 133
shot away, case of . . . xii 330
union of fractures of . . iii 562
Paterson, Dr., on corpora lutea . ix 570
on Rötheln at Leith . . x 285
Pathogenetic investigations, Remak's xxiii 503
Pathological anatomy, arrangement of iv 31
Dr. Vogel's . . xxii 323
elements of . . xxiii 64
essay on . . xix 362
generalization of . vi 301
humoral . . vi 297-98
influence of . . vi 293
its value . . i 400
of scurvy . . xx 147-49
organic . . vi 296
progress of vi 295, 297, 300
province of . . vi 298, 301
remarks on xiii 328, xviii 48
treatises on . . xvi 381
chemistry, essay on . . xix 364
treatises on . . xvii 424
history, Günsburg's . . xxiv 118
observations by Albers . . iii 220
by Goodsir . . . xx 289
periodic phenomena . . xviii 168
problems . . . . . vi 301
relation, principle of . . ix 463
researches . . . . . xxiv 135
Society of Dublin . . . viii 279
Pathology, a cause of its retardation xvii 474
Albers' observations in . . v 181
and physiology, Dr. Alison on xviii 213
and practice, on British . . ix 462

Pathology, and surgery, selections in . viii 512

therapeutics, Dr. Marx on xvi 472, 476

relation of . . xiv 197

Schönlein on . . xxi 20

special . . . xiii 327

a new phase in . . . xvii 473

basis of Dr. Jahn's . . . iv 122

cerebral, M. S. Pinel on . . xx 18

chemical, caution on . . xvii 449

clinical, Dr. Lanza on . . xxiii 47

comparative, Hensinger on . xxiv 81

difficulties of . . . . vi 108

difference in codes of . . vi 295

Dr. Alison's Outlines of . . xv 533

Dr. Emmert on . . . xvi 267

Dr. Fletcher's . . xv 367, 372

Dr. Jahn's new system of . iv 120

Dr. Richter's views on . . xv 411

Dr. Zimmerman's . . . xx 74

founded on physiology . . xiii 205

general, Dr. Marx on . . xvi 472

Dr. Schultz on . . xx 339

treatises on . . . xx 216

human, Mr. Mayo on . . iii 1, 55

humoral, remarks on . . vii 495

improvement of . . . vi 109, 111

increasing estimate of . . xvii 472

in relation to cerebellum . xxii 542

Mackintosh's principles of . iii 101

modern improvement in . . ix 464

M. Lisfranc's remark on . i 586

Mr. Walker's view of . . xiii 205

nervous, present limits of . iii 31

new researches in . . . xx 340

of brutes, observations on . xxii 35

the blood and inflammation xx 71

nervous system . . iii 2

Van Helmont . . . xvi 413

on education in . . . x 192

rational, Prof. Henle on . . xxiv 289

report on the progress of . xx 209

requisites and objects of . vi 107

scientific form of . . . xvii 475

special of Schönlein . . xxi 32

or empirical . . xxiv 294

theoretical, obstacles to a . iii 160

the science of abnormal life . vi 107

Patient, methodical examination of xx 310

method of examining a . . ii 476

on one judging for himself . viii 495

Patients, on kindness to . . xxi 448

Patissier, M., on the waters of Vichy xiv 309, 320, 347

Patriarchs, on the lives of the . xvi 510

Patriotism, false, of medical critics xxi 447

Patronage of quacks and impostors xxi 533

Pau, Mrs. Ellis on residence at . xiv 354

the baths at . . . . xiv 354

Pauli, Dr., his Observations in Surgery xxii 49

on reproduction of the lens . viii 247

Paul of Ægina, his midwifery . xiv 83

Paulus Æginetus, by Dr. Adams, Vol. II. . . . . . xxiii 251

his speculum vaginæ . . xiv 209

Pauly, M., his charges against Lisfranc xviii 32

Pauperism, effect of civilization on xviii 239

Pauper lunatics, asylums for . xix 319, 342

Paupers, on dietaries for . xvii 48, 49-51

Pavia, mortality in, in 1832-3-4 . i 250

Paving of towns, observations on . xxi 338

Payment of medical practitioners . xxi 265

Peacock, Dr., on obstruction of vena cava . . . . xxiii 410

Pearson, Dr., on broom seed in dropsy i 533

Pearson, Dr. G., his opposition to Jenner . . . . vi 487, 489

Peas, death from . . . . xiv 576

improvement of flour by . . xiii 491

nutritious properties of . . xiii 492

Pebble, or Brazilian-quartz, lenses . xvi 362

Pectinaceous principle of aliment . xvii 9, 13

Pectoriloquy, a sign of a cavity in lung iv 322

Dr. Harrison on . . . iii 554

remarks on . . . . i 483

value of as a sign . . . iv 322

Peculiarities, acquired, hereditary . xiii 167

Pedicular disease, remarkable . xii 432

Pediculi, remarkable formation of . xii 432

Peerage, duration of life of the . xxi 1, 9

Peken, his physiology and pyrethology i 606

Pelagian negroes, account of . . xxiv 469

or Oceanic races of mankind . xxiv 466

Pellagra, cases of at Padua . . x 208

Pelves, human, classification of . xxiv 79

Pelvic abscess, cases of . . . xxiii 291

abscesses, treatises on . . xix 559

cellular tissue, labour with tumours in . . . . . xi 504

encysted tumours, evacuating . xi 463

inflammation, with abscess . xviii 341

symphysis, lacerations of the . viii 51

synchondroses, destruction of xiii 118, 171

tumours, Dr. Lever on xvi 310, xviii 333

Pelvimeter, the finger and hand best iii 393, iii 412

the hand best . . . iii 412

Pelvimeters . . . . . i 106

Pelviotomy, operation of . . xv 247

Pelvis, congenital tumours of . . xiv 64

contracted, congenital? . . xi 171

on craniotomy in . . xix 417

deformed, natural delivery in . i 607

Prof. Martin's theory on . xxiii 280

yielding in labour . . xvii 536

deformities of, Hull's theory of iii 394

difference of, in races of men . xxiv 78

exostosis of, and embryotomy . vi 237

female, on exostosis of . . xii 490

in labour, former errors on the xi 167

malformation of . . . xvii 535

cases of . . . . xxiii 280

measurement of the . . viii 37

Dr. Davis on . . . iii 393

narrowed by fracture of sacrum xxiii 281

on obliquely contracted . . xi 167

case of . . . . xi 170

characteristics of . . xi 168

detection of . . . xi 173

diagram of . . . xi 169

Pelvis, obliquely contracted, origin of . xi 170

on the, in luxations of femur . iii 240

singular case of tumour in . xi 531

tumours in, obstructing labour xi 500

varieties of . . . . . xvii 275

Pemphigus, causes of . . . . xv 385

chronic, remarkable case of . xxiv 48

diagnosis of . . . . . xv 386

formation of . . . . . xxiv 122

in newborn infants . . vii 94

observations on . . . . xv 385

treatment of . . . . . xv 386

neonatorum, contagious . . xiii 533

Penances, religious, remarks on . iv 57

Penis, on amputation of the . . iii 266

new method of . . . x 269

sad effects of . . . . xiv 12

cancer of . . . . . . xxii 306

case of cancer of the . . . xiv 563

gangrene of, seldom fatal . ii 29

erectile tissue of the . . xix 509

structure of . . . . xiv 294

muscles of the . . . . xix 510

ornamental operation on . xxi 313

ossification of . . . . xx 136

Penitentiaries in Norway, account of xvii 88, 89

Pennock, Dr., on action of the heart ix 561

Pennsylvania, University of . . ii 584

Pension Society, Printer's, recommended . . . . . . i 285

Penwith hundred, account of . vi 3

Penzance in phthisis, remarks on . vi 23

Pepper, poisonous to fowls . . iv 130

Peppermint, use of in stomatitis . xxii 54

Pepsine, Wasmann's researches on . xvi 148

Perception and sensation distinct . v 503

import of the term . . v 503

meaning of the word . . xvi 173

suspended, strange case of . xii 565

Percival, Mr., his narrative . xi 104

on glanders and farcy . . xxii 35

Percussion, abdominal, neglect of . i 480

advantages of the finger in . iii 185

and auscultation . . . . xiv 173

art of medical . . . . xx 311

advantages of pleximeter in . iii 185

as a mode of diagnosis . . iii 543

back of fingers in . . . . iv 293

-caps, on pieces of, in the eye xvii 236

causes of resonance in . . iii 542-5

clavicular, in phthisis . . iv 321

Dr. Skoda on . . . . xiv 173

Drs. Williams and Clendinning on iii 543

effect of, on the deaf . . xxi 192

example of delicacy of . . vi 137

force of the stroke in . . iii 545

hammers and pleximeters . xiv 180

impeded by cambric muslin . iv 294

inattention to in England . i 479

in dilatation of stomach . . i 487

in infantile pneumonia . . vii 79

in relation to air in chest . iv 294

its discovery and progress . i 479

Percussion, mediate, on finical . xiv 181

mode of, in children . . iii 187

in phthisis . . . iii 188

performing . . . iii 545

using fingers in . . iii 187

not valued in Italy . . i 498

of the heart . . . . vi 132

of liver and lung, diagnostic in i 480

posteriorly . . . xx 199

scapular, in phthisis . . iv 321

sounds in . . . . . iii 544

use of caustic after . . vi 133

in diseased ovary . . iii 139

varying the force of . iv 294

Winterich's new hammer for . xiv 180

Dr. Günzburg on . . . xxii 232

Percy, Dr., on alcohol in the brain viii 539

Pereira, Dr., Materia Medica viii 353, 357, xiii 54, 83, xvi 352, 362

on food and diet . . . xvii 1, 54

Pereyra, M., his cases of phthisis xix 142

his diagnostic skill . . xix 144

on the cure of phthisis . . xix 140

Perforated spot in the brain . xviii 435, 437

Perforating ulcer of the stomach . xiii 516

Perforation by corrosive poisons . ix 374

in labour, Dr. Collins on . ii 77

remarks on . . . ii 77

internal, hemorrhage from . xxii 372

obstetric, Dr. Kilian on . . iii 421

Mad. Boivin on . . iii 421

Osiander on . . . iii 420

remarks on . . . . iii 420

scalpels in . . . iii 420

the trepan in . . . iii 420

of appendix cæci . . . iv 149

cæcum . . . . . ix 451

cranium, case of needless . iv 374

ileum from without . . xx 221

intestine and groin . . viii 279

intestines by worms . . ii 519

in typhoid fever, signs of ii 39

opium in . . . ii 61

works on . . . ii 62

membrana tympani . . iii 91

peritoneum . . . . ix 373

stomach and bowels . . xviii 534

stomach . . . . vii 243, ix 371

after death . . ix 376

by a worm . . xiii 563

by solution . . ix 375

case of iii 556, ix 55, xiv 248, 577

insensible . . . ix 375

suspicious . . . ix 373

ulcerative . . . ix 372

unique case of . . ix 373

Perforations by entozoa . . vii 224

spontaneous, of stomach . vi 221

Perforator, Prof. Naegele's described iii 420

Pericardial adhesions, effects of . xix 119

remarks on . . . xx 253

cystometer . . . xxii 211

Pericardiac effusions, epidemic . xx 151

Pericardial effusion, external signs of xix 118

percussion in . . . . xix 118

sounds in . . . . xix 119

exudations, structure of . . xxiv 119

sound of new leather . . xx 455

sounds, account of. . . . xx 179

Pericarditic effusions, paracentesis in xii 250

signs of . . . . . vii 368

Pericarditis . . . . . . viii 463

adhesions in . . . . . xxiv 558

age most liable to . . . . ii 320

and carditis, signs of . . . i 10

bruit de frottement in . . . xii 113

calomel and opium in . . . ii 321

cardiac bulging in . . . xxiv 553

chronic effects of . . . . ii 315

M. Bouillaud on . . . ii 319

case of . . . . . . . v 604

causes of . . . . . xxiv 566

course of . . . . . xxiv 559

creaking sound in . . . . xxiv 550

crumpling sound in . . . xxiv 551

diagnosis of . . . . . xxiv 548

difficult . . . . . ii 315

diagnostics of . . . . vi 134

disease of heart from . . . xvi 328

Dr. Latham on . . . . . xx 182

Dr. Roots on . . . . iii 150

Dr. Taylor on causes of . . . xxiii 415

Dr. Taylor on . . . . xxiv 548

effect of, on the brain . . . xii 333

effects of . . . . . xxiii 174

failure of diagnosis in . . xxiv 554

friction-sound in . . . . xxiv 549

from acute rheumatism . . . xxiv 567

Bright's disease xxiii 178, xxiv 569

metastasis . . . . xxiv 568

rheumatism, hypothesis on xxiv 569

general symptoms of . . . ii 318

heart-sounds in . . . . xxiv 551

hemorrhagic, account of . . i 259

in acute rheumatism . . . ix 485

brain-affections, treatment of xxii 427

children . . . . . . xvii 560

rheumatism . . . . xii 112

its connexion with rheumatism i 426

mercury in . . . . xx 192, xxiv 565

modes of death in . . . xxiv 561

morbid appearances in . xxiv 560, 561

mortality from . . . . xx 194

nervous affections in . . . xiii 456

on a subacute kind of . . . vi 389

peculiar aspect in . . . . xiii 456

phenomena of . . . . . xx 454

point in diagnosis of . . . xii 113

prognosis in . . . . ii 320

renewed, on signs in . . . xxiii 172

scorbutic, at Kronstadt . . . xii 250

sounds of the heart in . . . i 454

spasmodic disease from . . . ix 431

movements in . . . . xxiv 557

spontaneous cure of . . . xxiv 564

state of the blood in . . . xvii 433

Pericarditis, symptoms of . . xxiv 556

by Bouillaud . . . ii 316

tartar emetic in . . . . ii 229

tartrate of antimony in . . . ii 321

treatment of ii 320, xx 456 xxiv 564

valvular disease in . . . . xxiv 568

with dysentery . . . . xxiv 564

with effusion, diagnosis of . ii 328

without physical signs . . xxiv 555

wonderful cure of . . . . vii 72

Pericardium, adhesions of the . iv 42

case of hemorrhage into . . v 607

hernia of . . . . iv 532

congenital absence of . . . ix 440

dropsy of, position in . . xii 281

dryness of . . . . . xxiv 560

effusion into, signs of . . xiii 455

fatty matter under . . . vi 47

morbid anatomy of . . . iv 41

thickening of . . . . iv 42

sanguineous effusion in . . . xii 250

white spots on . . . . vi 47

Perilymph of internal ear . . viii 79

Perimysium, or sarcolemma . . xxiv 130

Perineal laceration, danger of, in shoulder presentations . ii 75

Perinephritis, diagnosis of . . xv 477

M. Rayer on . . . . . xv 477

nature of . . . . . viii 150

Perineum, and bladder, Dr. Monro on xiv 540

central laceration of . . . v 580

Dieffenbach on rupture of . vi 536

effusion of urine in . . . x 368

lacerated, M. Roux on . . xxiii 283

laceration of, cured . . . viii 284

ring for . . . xiii 549

layers of fat in . . . . x 367

Mr. Morton's anatomy of . viii 244

on supporting the . . . iii 413

in labour . iii 407, xii 470

operation for lacerated . . viii 284

passage of a child through . xix 427

ruptured, consequences of . vi 536

Dieffenbach on . . . iii 423

isolation of . . . . iii 423

Prof. Kilian on . . . iii 422

treatment in . . . iii 423

rupture of, Dieffenbach on . xxi 323

in labour . . . . xiv 369

reuniting . . . . xi 195

structures in the . . . . x 366

support of, mode of . . . xxi 87

surgical anatomy of . . . x 366

Periodeutic physicians . . . xxiii 91

Periodical maladies, Prof. Manni on viii 57

Periodic cycles . . . . . xviii 172

movements, Dr. Laycock on . xii 63

pathological phenomena . xviii 168

return of epidemics . . xviii 172

vital phenomena . . . xviii 166

Periodicity, Dr. Cowan's definition of iii 162

in disease . . . . xx 216, xxiv 317

diseases, remarks on . . xxiii 588

Periodicity in fevers, Dr. Laycock on xviii 168
in health and disease . . xviii 165
of intermittents, cause of . xviii 235
remarks on the law of . . viii 505
Schwann and Quetelet on xviii 165, 177-8
Periods, esoteric causes of . . xviii 171
of years, determination of . xviii 173
vital, the causes of . . xviii 169
Periosteum, and medullary membrane,
identity of . . . xxiv 404
formation of bone by . xxiv 395, 401
forming osseous substance . xx 306
inflammation of the . . ii 536
Mr. Syme's experiments on . viii 285
power of, to form new bone . viii 285
report on . . . . . xxi 548
Periostitis, mistake of Sir C. Bell on vi 160
Peripheric nervous influence, what iii 209
Peripneumonia notha . . . xx 442
Peristaltic persuaders, formula of . xix 31
Peritoneal inflammation, products of xxiv 120
Peritoneum, diagnosis of adhesions of ii 167
in intestinal perforations . vii 248
morbid anatomy of . . iv 46
changes in . . . ii 167
nervous affection of . . vi 356
perforation of . . . . ix 373
state of, in scirrhous liver . iii 76
tuberculated, remarks on . iv 49
Peritonitic effusion, virulence of . xxii 197
Peritonitis, adhesions from . . iv 48
calomel and opium in . . iii 149
cancerous, diagnosis of . . xvi 444
chronic, in children . . xxiv 47
in phthisis . . xvi 436, 443
in relation to phthisis . v 157
constipation in . . . iii 75
diagnosis of . . . xxi 96
effusion of lymph in . . xiii 114
effusions in . . . . iv 47
erysipelatous . . . ii 25
from injections into uterus . x 566
from perforation of stomach, case of xxiii 126
hysteria simulating . . xvii 135
in newborn children . . xxiii 301
the fœtus . . . vii 284
typhoid fever, almost always fatal ii 39
Vienna, nature of . . xxiii 607
left to nature, case of . . xxii 560
morbid appearances after . iv 50
new signs of . . . xi 234
treatment of . . . xi 234
resembling pregnancy . . xx 526
simulated by hysteria . . xii 107
singular case of . . . xxiv 121
strumous . . . . . xx 240
tubercular chronic . . iii 76
tuberculous, symptoms of . xvi 443
two kinds in women . . ii 25
with effusion through navel . xiv 549
Perityphlitis, description of . . xviii 313
of Albers, doubtful . . ix 456
Perkinism in rheumatism, account of xiv 420
Permanent contraction of fingers . iv 169
Permeability of tissues, physiological viii 322
Permeation of fluids in the body . xxiv 276
Perplication, arresting hemorrhage by viii 327
PERRY, Dr., his letter on typhus fever iii 548
his sanitary report . xviii 492, 505
PERRY, Mr., on varicose aneurism . iv 138
Persia, fainting fever in . . xvi 558
Persian doctor, consultation with a iv 387
epidemic, curious, case of . xvi 561
fainting fever, bleeding in . xvi 563
history of . . xvi 558
pathology of . . xvi 561
treatment of . . xvi 561
varieties of . . xvi 559
Perspiration, checked, morbid effects of xvii 484
cure of diseases by . . xxii 450
cutaneous, report on . . xix 567
hectic, pus said to be in . i 509
in the horse . . . . viii 492
obstructed, treatment of . ii 229
remarks on . . . . . xx 312
spontaneous in diseases . . viii 491
suppressed, a cause of dropsy . ii 226
the cause of diseased kidneys ii 226
two conditions of . . . viii 492
processes of . . . viii 490
Perspiratory tubes, description of . xxi 199
Perth, medico-legal case at . . iii 271
Pertussis, acetate of morphia in . ii 178
case of, treated with morphia ii 179
fumigations in . . . i 574
subcarbonate of iron in . . vi 222
trial of many remedies in . ii 178
Peru, Dr. Smith on the diseases of . x 285
on the diseases of . . . x 583
Peruvian skull—Aymara race . xviii 381
Chincha race . xviii 380
Huanca race . xviii 382
present time . xviii 382
skulls, and phrenology . . x 483
interparietal bone in . xviii 384
Peruvians, buildings and roads of . x 480
form of their skulls . . xviii 380
skulls of the ancient . . x 478
subjugation of, by Pizarro . x 481
PESCHEL, M., his Elements of Physics xxii 553
Pes elephantiacus, case of . . iv 227
equinus, and varus, treatment of iv 231
relative frequency of . xiii 8
treatment of . . . ix 229
Pessaries, Lisfranc on the use of . xviii 27
modifications . . . xvii 544
Prof. Dieffenbach on . . iii 512
use of, in prolapsus uteri iii 134, xix 356
when very injurious . . ii 512
Pessary, discussion on the use of the iv 182
lever of Dr. Sander . . xx 541
use of the . . . . xxi 86
Pestilence, conduct of medical men in xxi 449
Pes varus, division of tendons in . ix 229
Petechiæ in fever, remarks on . ii 40
Petechial cowpox, case of . . xv 154

Petechial typhus prevalent in wars . xi 28
Peter de Apono, account of . . xix 124
Petersburg, foundling hospital of . xii 273
Petersen, M., his writings of Hippocrates . . . . . xvii 450
Peter the Great, a medical student i 603
his practice of surgery . i 603
Petit, M., on alkaline mineral waters xiv 350
on the waters of Vichy . xii 388, 394
Petrarch, tranquil death of . vi 497
Pétrequin, M. on amaurosis . xvii 155
on artificial auscultation . vi 532
on medico-chirurgical anatomy xx 133
Petrifaction, artificial, of animals . ii 532
of animals, Segato on . . ii 532
of the tissues . . . ix 246
Petrous nerves, Dr. Bidder on the . vi 204
Petromyzon marinus, spinal cord of the v 294
Pettigrew, Mr., distich on his work vii 226
his Medical Portraits . . ix 543
his Portrait Gallery . vi 194, vii 225
on medical superstitions . xviii 234
Petzholdt, Dr., on smallpox . v 469
Peyer's and Brunner's glands in typhus viii 431, 446
Peyer's glands, anatomy of . . vi 218
Chomel on lesions of . xii 28
corpuscles of . . i 523
course of blood-vessels in i 523
dissection of typhoid . viii 446
general anatomy of . i 522
in infants . . . . i 525
in man . . . . i 524
hard patches in . . xii 27
lesion of, in cholera . xii 299
in typhus xii 299, 300, 311, 316, 325
Louis on lesions of . . xii 28
minute structure of . . i 236
on fever in relation to . xi 420
structure of . . . xiv 291
typhus, from lesion of . xii 294
patches, remarks on . . v 184
Pezzoni, Dr., on cases of plague xvi 290, 293
on the plague . . . xv 152
Pfeffers, waters of . . . . xiv 351
Pferdemanke in the horse . . ix 79
Phagedena, fatal, from blister on knee ii 15
Phagedenic sloughing, and ulceration xi 321
ulceration, remarks on . . xii 339
venereal ulcer, forms of . . x 405
mercury in . . x 407
Phanerogamia, reproduction in . vii 183
Pharmaceutical chemical errors . xvi 203
explanation, important . . xi 441
Society, account of . . xiii 211
Pharmaciens in Belgium . . i 608
Pharmacologia, Dr. Paris's . xvi 188, 205
Pharmacopœia, composition of a . xiii 54
London, for 1836 . . . iv 101
of the United States . xiv 437, 441
the Prescriber's (2d edit.) xii 221, xv 227
Pharmacopœias, nomenclature of xiii 56, xvi 189
on the language of . . . xiii 56
various . . . . . xiii 54
Pharmacy, American Journal of . ii 233
of the ancient Hindoos . . xxiii 536
works on . . . . . xiii 54
Pharyngeal carcinoma . . . xxii 295
Pharynx, abscess at back of the . xi 411
and larynx, anatomy of . . xxiv 483
bursa in the . . . . xvii 258
follicular disease of . . . xxiv 485
nervous affections of . . iv 135
sacculation of the walls of . xxii 295
Pharyngitis, scarifications in . . vii 551
Phelan, Mr., on Irish medical charities ii 183
Philadelphia prison, system in . xii 500
Philadelphia, typhus fever of . . xii 294
Philanthropos, on Bethlem Hospital xiii 215
Philanthropy, effect of civilization on xviii 244
Philiatria, the, of Copenhagen . ii 297
Philip, Dr. W., his electro-neurology xv 525
his Gulstonian lectures, 1835 . ii 129
on affections of the brain . . ii 129
on indigestion . . . xv 524
strictures on his style . . ii 147
Philip of Macedon, and Menecrates xix 122
Phillipp, Dr. P. J., on diseases of the chest iii 184
Phillips, Dr. C., his philippics on Guerin xiii 24
on Dieffenbach's surgery . . xi 194
stammering . . . xii 209, 211
subcutaneous tenotomy . xiii 1, 23
Phillips, Mr. B., his treatise on Scrofula . . xxii 124, 155
on cauterizing the urethra . xv 366
fractures of the atlas . . iv 141
ovarian operations . . xx 101
prolapsus uteri . . . viii 596
results of amputation . . vi 561
section of tendons . . viii 596
tumours in the neck . . xv 155
Phillips, Mr., his London Pharmacopœia xiii 54, 88
his Pharmacopœia . . . iv 101-3
Phillips, Sir C., on strabismus . xi 474, 492
Philocophus, by J. B. Bulwer . viii 69, 71
Philological delinquencies . . xvi 492
Philosopher, character of the true . xiii 168
spirit of the true . . . ix 428
the true and selfish . . ix 431
Philosophers, jealousies and disputes of ix 431
lack of philosophy in . . xii 208
Philosophia Prima of Bacon . . ix 424
Philosophical Transactions for 1835 ii 212
Philosophic spirit, Dr. T. Brown on a ix 427
Philosophising physicians of nature . xv 462
Philosophising, principles of . . ix 424
Philosophy, ancient and modern systems of v 82
Bacon's definition of . . v 82
benefit from systems of . . v 319
its use in medical science . i 368
medical, Dr. Bouillaud on . iii 384
Mr. Powell on the inductive . v 548
natural, Dr. Bird's . . ix 529
of the Greeks . . . xvii 458
value of . . . . ix 427
Philosophy of Health, by Dr. S. Smith i 313, v 380
of Living, by Mr. Mayo . . v 380, 383

Philosophy of Living, Dr. Ticknor's v 381, 409

of medical science . . . xx 140

of medicine . . . . . xxiii 39

spiritualist school of . . xxiii 99

Philoxenus an oculist . . . xxiv 364

Philumenos, midwifery rules of . xiv 83

Phlebitic ophthalmia . . . . x 33

Phlebitis hepatica neonatorum . x 580

Phlebitis, diagnosis of . . . xii 431

from angioleucitis ii 557

Dr. Roots' observations on . ii 487

fatal case of . . . . vi 228

intra-cranial, case of . . xx 229

M. Lisfranc's treatment of . i 496

puerperal, cause of . . . xiii 115

œdema in . . . xiii 118

treatment of . . . xiii 120

pulmonary, case of, by Dr. Lee ii 163

remarkable case of . . . xiv 53

subacute, not serious . . iii 68

uterine, characters of . . xiii 104

deposits of pus in . xiii 105, 118

diagnosis of . . . xiii 105

in puerperal fever . xxi 276

on the origin of . xiii 106, 115

prognosis of . . . xiii 106

Phlegmasia dolens, nature of . . xiii 119

not from phlebitis . xii 114

remark on . . xxi 92

Phlegmon, and erysipelas, distinction of xi 326

use of cold water in . . iii 118

iodine in . . . viii 521

Phlegmonoid suppuration . . ii 24

Phlogistic diseases, Dr. Chiappa on . i 250

Phocomeles, genus of monsters . viii 6

Phonation, action of Willis's nerve on xiii 226

Phosphate of lime urinary deposits . xvi 316

Phosphates, Dr. Prout on deposition of xi 356

in the urine, source of . . xx 68

mixed, deposition of . . xi 356-7

necessity of, in manures . . xvii 226

Phosphatic urine, a remark on . vii 163

case of . . . xx 68

diet in . . . xi 357

from nephritis . . viii 155

treatment in . . xi 357

use of narcotics in . xx 69

Phosphorescence of organized beings xxiii 395

Phosphoric acid, diluted, the dose of iv 110

use of, in Germany . xix 241

in phthisis . iv 110

Phosphorus, a dog poisoned by . i 245

as an element of food . . xvii 3

poisoning by . . . . . xviii 536

Phrenic nerve, *figure of* . . i 561

origin of, traced . . i 560

nerves, effects of tying the . iii 155

rupture, diagnosis of . . xxiv 172

Phrenitic symptoms, Dr. Abercrombie on iii 305

remarks on . . iii 305

Phrenitis, contraction of pupil in . iv 491

Dr. Hope on . . . . xii 100

simulated by hysteria . . xii 107

Phrenological developments, taking xiv 70

divisions of the brain . . ix 207

Journal . . . . . . ix 193

system, doubts on . . . xxii 519

Phrenologists, in London, Dr. Marx on xv 23

on failures of . . . . ix 203

Phrenology, anatomical remarks on . xviii 432

and Peruvian skulls . . x 483

arguments in favour of . . ix 209

Cuvier favorable to . . ix 200

definition of . . . . ix 197

Dr. Gordon's attack on . . ix 192

Dr. Holland's objections to . ix 199, 203

faults and errors in . . xxii 543

founded in truth . . . xx 34

frontal sinus in relation to . ix 205

Gall's first idea of . . . ix 196

leading merit in . . xiv 73

method in . . . xiv 73

importance of, if true . . ix 191

inaccurate observations in . xxii 523

in relation to insanity . . xx 34

its nature and uses . . . xxii 230

leading propositions in . . xiv 66

metaphysical deficiencies in . xxii 527

mode of studying . . . ix 206

obstacles to progress of . xiv 68, 70, 73

of criminals . . . . xiii 527

on organic size in . . . xiv 76

passing remarks on . . vii 14

precautions in studying . . ix 212

present condition of . . xiv 67

state of . . . . . ix 192

popular, consequences of . . xiv 69

extravagance of . . xiv 69

principles of . . . . . ix 197

proper method of studying . xiv 80

proposed modification of . . xxii 518

sources of fallacy in . . xxii 524

Spurzheim's method in . . xiv 72

the skull in relation to . . ix 205

true and false . . . xiv 65

true, utility of . . . xiv 70

useful applications of . . ix 215

views at variance with . . xxii 517

works on . . . . . . ix 190-1

Phreno-magnetic phenomena, key to xix 482

Phosphatic diathesis, origin of . viii 155

Phthisical complexion, remarks on . xiv 193

constitution, remarks on . . xxiii 438

habits, Van Diemen's Land for . vi 23

hygiene of the . . . . xx 108

sputa, filaments in . . . iv 318

subjects, outward aspect of . xiv 192

Phthisical, treatment of the . . xvi 253

Phthisis, accidental, Dr. Stokes on . iv 323

acute, description of . . xvi 447

adult and infantine, compared . xx 98

a general law in . . . xvi 437

almost invariably fatal . . xvi 452

American statistics of . . iii 526

anatomical stages of . . xvi 427

and ague, antagonism of xx 217, xxii 212, 214

Phthisis, and bronchitis, diagnosis of xx 447

chronic bronchitis, on . xxiv 505

emphysema, diagnosis of . xx 448

scrofula, connexion of . xviii 486

contrasted . . . xix 22

deaths from . . xxii 135

identity of . . xxii 135, 137

independence of . xix 22

animals infected with . . viii 249

Aretæus on . . . . . iv 491

arsenical cigars in . . . xii 249

asses' milk in, M. Louis on . xvi 458

at Martinique . . . xviii 158

auscultic sounds in . . iii 188

average duration of . . xviii 305

benefit of sea-voyages in . . . xv 14

bronchial, diagnosis of . . xvii 309

in children . . xvii 305, xx 99

symptomatology of . . xvii 308

calcareous expectoration in . xvi 440

cases for diagnosis in . . ix 338

cases of, British Guiana for . i 508

cause of, in Englishwomen . xi 425

causes of . xvi 452, xx 102, xxiii 437

cauterization of throat in xxiv 401, 503, 505

chalybeate drink in, at meals . xvi 458

change of climate in . . xxiv 109

chlorine gas in . . . . iv 212

chronic, signs in . . . . ix 337

chronic peritonitis in . xvi 436, 443

cicatrization in . . . . ix 340

clinical difficulties in signs of . ix 335

common salt in . . . . xvi 455

comparative site of . . . ix 334

conditions of the tongue in . xvi 441

confounding diseases with . iv 322

constitutional, Dr. Stokes on . iv 323

contagiousness of . . . . ix 339

contractions of chest in . . iv 322

course and progress of . . ix 337

crackling rhonchi in . . ix 328

creosote in . . . . . xvi 456

crumpling sound in . . . ix 328

curability of . iv 323, ix 323, 340, xix 140

cure of . . . . . . xvi 452

by cod-liver oil . . xix 141

by naphtha . . . xvi 490

list of drugs, &c. for . xix 141

curious tale of infectious . ix 340

cutlery-grinders', symptoms of xviii 305

deafness in . . . . . xvi 446

deaths from, in England . ix 354

decumbiture in . . . . xvi 439

diagnosis of . . . . . xvi 450

from bronchitis . . iv 320

diarrhœa in . . . . . xvi 442

digitalis in . . . . . xvi 456

the hectic of . . . v 189

diseased follicles in . . . ii 46

diseases which suspend . . xix 22

Dr. Bene on . . . . . i 22

Dr. Chapman on . . . . xix 22

Dr. Evans's lectures on . . xxii 85

Phthisis, Dr. Graves on . . xvi 249

Dr. Guy on the pulse in . . ix 337

Dr. Hall's alcoholic lotion for xxiii 187

Dr. Prout's urinary theory of xi 335

Dr. Stokes on varieties of . iv 322

duration of . . . . . xxiii 436

M. Louis on . . xvi 438, 447

dyspeptic, bath for . . iv 254

dyspnœa in first stage of . ix 333

early, expansion of chest in . i 480

Dr. Stokes's diagnosis of iv 320

effects of, on cardiac disease . xxiii 178

on the heart . . . vii 143

emaciation in . . . . xvi 446

emetics in . . xvi 456, xviii 160

essential cause of . . . xvi 251

etiology of . . . . . xiv 225

exemption from, in the navy . xv 13

expectoration in . . . xxiv 127

fatty liver in . . . . . iii 77

M. Louis on . xvi 435

ferruginous medicines in . . xvi 458

fibrous bands in lungs in . xxiii 435

fistula in ano in . . . xvi 434

Florida as a residence in . . xvi 42

formed, on tonic drinks in . xvi 457

frequency of pleurisy in . . xix 113

frequent in the insane . . v 375

from albuminous deposit . iv 357

grinding cutlery . . xviii 304

gritty particles . . xx 251

hæmoptysis in xvi 440, xviii 159, xix 116

hereditary influence on . . xvi 453

improved knowledge of . . xvi 459

in America, change of climate in xiii 428

army and navy . . . xv 11

captive animals . . . xx 104

children, observations on xx 554, 558

pneumonia in . xvii 310

report on . . xvii 559

incipient, curable, forms of . iv 323

mercury in . . . iv 323

treatment of . . iv 323, xii 331

increase of viscera in . . vii 143

in different classes . . . xx 423

Great Britain and Malta . xiii 434

India, remarks on . . iii 330

males and females, table of . xvi 319

relation to catarrh . . ix 329

countries . . xiv 44, 46

marshy districts xxii 211-15

menstruation . . xiv 395

occupations . . xx 426

Sweden on the increase . xxiii 429

upper part of lungs . . xvi 319

western tropics . . . xiii 426

West Indies . . . viii 219

influence of climate on . . xxiv 107

employment on . xx 214

season on . . xxiv 105

stays on . . xvi 453

inhalation in, of conium, &c. . v 606

of chlorine in . . . xvi 456

Phthisis, inherited or acquired . ix 324

intestinal cicatrization in . xvi 433

iodine, chlorine, and tar in . iv 324

issues in, disapproved of. . xvi 458

its prevalence in factories . xv 310

laryngea, remarks on . . iii 72

Sir C. Bell on . . vi 165

laryngeal . . . . . v 432-33

causes of . . . v 433

fatality of . . . xvi 247

four species of . . v 433

observations on . . xviii 293

latent, M. Louis on . . xvi 449

little affected by temperature . xxiv 108

local limitations of . . xiii 430

localities in relation to . . vi 22

location of . . . . . xvi 317

louder expiration in . . iv 321

Louis on, new translation of . xviii 526

low ratio of, in Birmingham . vi 23

lung oftenest affected by . xvi 318

male sexual passion in . . xvi 445

march or course of . . xvi 447

marsh miasm adverse to . xiv 44

medicated inhalation in . . xix 23

menstruation in . . . xvi 445

mesenteric, of children . . xvii 311

milk and vegetable diet in . v 189

mode of percussion in . . iii 188

morbid anatomy of . xv 89, xvi 425

mortality from . . . xvi 438

in cities, table of . xxiv 106

in England . . xi 425

most fatal in spring . . xxiv 106

movements of chest in . . ix 330

moxas below clavicles in . xxii 469

M. Fournet on diagnosis in . ix 338

on the causes of . . ix 324

M. Louis' researches on . i 165, xvi 425

M. Pereyra's cases of . . xix 142

naphtha in . . . . . xix 146

new mode of using iodine in . xii 282

new theory of . . . xvi 227

night perspiration in . . xvi 441

nitrate of potash in . . xix 23

number of deaths from . . xx 428

observations on . . . xii 349

of children, Dr. Green on . xx 97

on ardent spirits causing . xiii 429

other diseases as causes of . xxiii 438

oxygen gas in . . . xix 233

palliative treatment of . . iv 324

pathology of . . . xxiii 421, 432

peculiarities of stomach in . xvi 432

peculiar voice in . . . ix 329

perforation of lung in . . xvi 447

phosphoric acid in . . . iv 110

physical condition in . . xix 114

diagnosis of . . . xvi 451

signs of, Dr. Stokes on . iv 320

pleuritic lesions peculiar to . xvi 430

pleurisy in, incurability of . xvi 445

pneumonia in . . xvi 430, 444

Phthisis, pregnancy arresting . xvi 445

prevalence of, in the army . viii 225

prevention of. . . . xx 108

and cure of . . xxiv 491, 505

by diet, &c. . . . ii 387

M. Louis on . . . xvi 457

profuse expectoration in . . xvi 440

prognosis of . . . . xvi 452

proper diet in . . . xvi 458

protoiodide of iron in . . xvi 454

prussic acid in . . . xvi 456

pulmonalis, early signs of . vi 534

epidemic . . . vi 227

Rokitansky on . . xv 90

pulmonary, in children . . xvii 310

pulse in . . . ix 337, xix 144

rapid, cases of . . . xvi 448

mercurialization in . . xvi 253

rare in Van Diemen's Land . vi 20

rarity of, in China . . . xxii 186

relative prevalence of . . vi 22-4

remissions in . . . xviii 212

respiration in . . . vii 263

first stage . . . ix 327

retraction of thorax in . . ix 332

rhonchi in first stage of . . ix 328

rife among black troops . . vi 150

sal ammoniac in . . . xvi 455

scrofulous inflammation in . xvi 250

signs of first stage of . . ix 327

irritation in . . . iv 320

obstruction in . . . iv 320

Sir J. Clark on . . . . i 70, 84

smallness of the heart in . xvi 432

specifics for . . . . xvi 457

sputa in . . . . xvi 439

state of intestines in . . xvi 433

statistical researches in . . xiv 225

statistics of . . . . xviii 499

and treatment of . . xx 250

stout children often born in . iv 449

subcarbonate of potass in . xvi 455

subclavicular sign in early . ix 333

supplementary vessels in . xvi 429

symptomatology of . . xvi 438

table of physical signs of . ix 335

seasonal influence on . xxiii 436

tractableness of, Dr. Evans on xxii 88

treatises on the cure of . . xix 140

treatment of . . xvi 253, xxiii 437

M. Louis on . . xvi 454, 457

tubercular, Dr. Gellerstedt on xxiii 429

M. Rayer on . . . xx 219

ulcerations of air-passages in . xvi 431

unknown on coast of B. Guiana i 508

use of the pure alkalies in . xiv 193

varying expectoration in . xvi 439

vibratile motion of chest in . ix 330

vomiting in, Dr. Seymour on . xxiv 144

warmer climate in . . . xvi 458

when fatal in first stage . . i 22

without expectoration . . xvi 440

Phycomater of plants . . . ix 502

Phymosis, Malgaigne on treatment of xxiii 155

Physiatrix system of medicine . xxiii 594

Physic, ancient Hindoo practice of xxiii 540

Dr. Mackintosh's practice of iii 101

Dr. Watson's practice of . xvii 119

fallacy of the art of . . iii 469

practice of, reformation in . xxi 262

works on the practice of . ix 461

Physical and vital properties . . v 324

diagnosis, on comparison in . iv 296

forces, on correlation of . . xxiii 247

history of mankind . xxiv 49, 441

inquiries, Prof. Daniel on . ix 422

phenomena of living beings . xxiii 377

science, Mr. Smee on . . xvii 517

Mr. Smee's summary of . xvii 517

use of, to medical men . v 317

sciences, importance of . . ix 420

signs and symptoms, remarks on iv 288

detection of disease by . iv 296

Dr. Gerhard on . . iii 186

Dr. Williams on . . xvii 499

use of, before operations . ii 7

value of . . . . . iv 288

partial . . . iv 296

substrata of Dr. Laycock . xxiv 186

Physician, animating principle of . xxi 441

deserts and duties of . . xxii 115

his peculiar difficulties . . xii 8

his *saper-fare* or *savoir faire* . xxii 117

Life of a Travelling . . xvi 265

mental elevation of the . . xix 121

of a family, duties of . . xxi 506

qualities for a successful . . xv 27

the art of the mental . . ix 189

the true and counterfeit . . xvi 198

Physicians, ancient, of Western Europe xxiii 96

and druggists, connexion of . x 220

and surgeons, education of xiv 407-8

atheism and irreligion of . xxii 120

benefit from travel to . . xxi 170

College of, new charter of . xix 552

credulity of . . . . . viii 483

elderly, sarcasm on . . xi 499

English, Dr. Marx on . . xv 27

for the poor, remarks on . xiv 407

notions of the Hindoos on . xxiii 534

of asylums, duties of . . xix 601

of Prussia, their examination . i 300

paucity of eminent . . . vii 115

periodeutic . . . . xxiii 91

predicating sect of . . . xxiii 39

under the letter A . . . xix 553

Physics, M. Peschel's Elements of . xxii 553

Physiognomical rugæ of the face . xx 313

semeiology of infants . . xi 201

Physiognomy, medical, remarks on xii 108

of children in diagnosis . . ii 356

Physiological acts, mean result of . iii 41

anatomy, on the study of . x 180

discoveries by Dr. Todd . . iv 281

Essays of 1843-4, list of . xix 593

hypotheses, remarks on . . v 327

Physiological inquiry, objects of . v 331

objections to phrenology . xxii 519

principles of Borelli . . i 383

practitioners, training of . xxi 530

readers, on sneers at . . xxi 529

researches, Dr. Davy's . . xii 129

Mr. Shaw on . . . xiv 568

Nasse's . . . . iv 414

state of a patient . . . xxiii 41, 48

teacher, disease the best . . xxiii 171

Physiologist, aids of . . . v 331

investigations of . . . v 333-36

perplexities of . . . v 328, 336

requisites of a good . . v 318

Physiologists, a class of, described . iii 465

a genus irritabile . . . v 621

disputes of . . . . iv 78

errors of . . . . viii 309

Physiology, aids from chemistry in xxi 118

ancient Hindoo . . . xxiii 533

and anatomy of man . . xv 537

report on xxi 541, xxii 261

intellectual philosophy . xv 535

natural history, connexion of v 331

pathology, Dr. Alison on xviii 213

theories in . . v 317

therapeutics, relation of . xiv 197

an inductive science . . v 326

animal, Dr. Carpenter on . xvii 241

basis of . . . . . xv 143

best mode of studying . . v 340

chemical school of . . . i 383

chemical, Sylvius father of . i 383

comparative, of Tiedemann . iv 5

considerations on . . . iv 5

difficulties in studying . v 328, 336

Dr. Alison's Outlines of . . viii 241

Dr. Bostock's System of . iii 477

Dr. Carpenter's Manual of . xxi 480

Principles of . . xix 245

Dr. Dunglison's . . . vii 229

Dr. Griscom on . . . ix 537

Dr. Oliver's First Lines of . vi 195

Dr. Roget's Outlines of . . ix 534

Drs. Arnold on . . . viii 476

dynamic school of . . . i 384

education in . . . . x 183

of youth in . . . vii 214

effect of Bichat's researches on i 388

error of Mr. Whewell on . v 328

evil spirits retarding . . xiv 491

experiment in, uncertain . . xi 277

final causes in . . . v 338

First Lines of, Dr. Oliver's . xiii 222

for the use of schools . . ix 535

foundation of . . . . xiii 160

general, Dr. Carpenter on . xiii 160

observations on . . xiii 160

German school of . . . v 82

writings on . . . v 77

hint for a popular book on . vii 216

human, Dr. F. Arnold's . viii 476

Dr. Carpenter's xiii 467, 486, xxiii 245

Physiology, human, Dr. Dunglison's xviii 530

Dr. Elliotson's . . xi 497

indebted to Aristotle . . i 380

in relation to medicine . . xx 177

to philosophy . . . v 86

to theology . . . v 339-40

mathematical school of . . i 383

merits of various treatises on . xiii 468

method of pursuing it . . v 321

Mr. Carpenter's new work on vi 590

Mr. Carpenter's . . vii 168, 185

Mr. Paget's report on xiv 259, xvii 249

Mr. Walker's notions on . xiii 207

Mr. Whewell on . . . v 326

M. Sebastian's Elements of . xxi 282

Müller's Elements of . . ix 244

of Aristotle and Galen . . i 381

Boerhaave . . . . i 386

Haller, Dr. W. Clark on . i 386

Hippocrates . . . i 380

Hoffman . . . . i 386

Paracelsus . . . . i 383

Stahl, main error of . . i 385

the ancients . . xv 442, 452

the brain, Mr. Noble on . xxii 488

the thymus gland . . xx 159

on observation in . . . v 331

on philosophy in . . . v 338

on works of popular . . ix 536

pathological, Dr. Lebert on . xxii 18

popular progress of . . xvii 531

works on . . . vii 214

present progress of . . i 389

prospects of . . xiv 492

state of . . . xiii 469

principles of, by Dr. A. Combe i 313, 328

progress of, in England . . v 98

recent advance of . . . xiv 494

report on the progress of xix 249, 563

researches in . . . . xxiv 128

special, of Van Helmont . . xvi 408

still in its infancy . . . v 320

the science of normal life . vi 107

thoughts applicable to . . v 320

Todd's and Bowman's . . xx 201

treatises on . . . ix 534, 537

use of the balance in . . v 332

microscope in . . v 333

theory in . . . v 87

utility of, to artists . . xviii 442

various treatises on . . v 75

vegetable, an axiom in . . iv 14

remarks on . . . iv 2

report on . . . x 248

Wagner's Elements of . . xi 507

Whytt's Principles of . . i 385

Physometra, case of . . xiii 246, xix 355

Dr. Meigs on . . . xxi 88

of the uterus, Lisfranc on . xviii 19

Phytolacca decandria, in rheumatism xxi 372

Phytologist, the . . . xxiv 436-37

Phytophagous doctrine, arguments for xxi 151

Pia mater, inflammation of . . xxii 407

Pica, remarkable case of . . iv 222

Picrotoxine, nitrogen discovered in xiv 518

Pigeon-breast, Dupuytren's method with iii 174

Mr. Coulson on . . iii 174

Pigmentary membrane, a nonentity ii 155

Pigment, Mondini's membrane of the ii 352

-cells and granules . . . xiv 265

observations on . . xxiv 69

Pigs, keeping, in towns, nuisance of xxi 341

slaughterhouse for, in Manchester xxi 354

Pig's feet quickly digested . . xiii 494

Pike, seminal corpuscles in . . xiii 232

Pilcher, Mr., on the ear viii 68, 70, xiii 517

Piles, a cause of hemorrhage in . xii 116

aperients in . . . . . x 46

different species of . . v 482

dyspepsia from . . . xix 28

internal, bleeding arterial in . xii 117

M. Lisfranc's treatment of . xviii 34

Mr. Mayo on . . . iii 76

nature and treatment of . . xxi 368

ointment for, by Dr. Geddings i 579

use of nitrate of silver in . xiii 62

Pill-box, hydatid, description of . xvii 195

Pill, dinner, of Dr. Burne . . x 47

Mr. Mayo's dinner . . . v 390

-taking by young ladies, evils of v 391

Pills made with copaiba, inactive . iv 117

mistaken for gall-stones . . xvi 204

observations on . . . xvi 204

refractory, mistaken for gall-stones iv 117

Pilulæ colocynthidis ferrosæ . . xiii 85

Pimelosis hepatica, Dr. Albers on . viii 554

Pince à faux germe, Dr. Dugès on vi 86

Pineal gland, dropsy of the, case of vi 268

Pin, curious passage of a . . xiv 396

new, for lacerated sphincter ani iv 469

pulmonary disease from swallowing xix 30

swallowing, curious case of . x 588

Pinhole pupil in typhus, treatment of xv 466

Pinel, and Broussais, war between iii 387-8

his Nosographie Philosophique iii 387

problem enunciated by . . iii 387

tribute of praise to . . xix 297

unchaining the lunatics . . i 286

Pinel, M. S., on cerebral pathology xx 18

Piorry, M., his facile princeps in percussion . . . vi 138

his medical nomenclature . vi 128

his researches on percussion . i 479

on diagnosis . . . vi 128

Piper angustifolium, or matico . xvii 100

Piperine, use of, in ague . . ii 178

Pirondi, Dr., his ludicrous errors . viii 534

his travels in England . . viii 534

Pitcairn, Dr., case of epistaxis in x 61

problem enunciated by . . iii 387

Pitchcap for porrigo, remarks on . ii 153

in favus, remarks on . . xv 123

Pitchers in plants, remarks on . iv 14

Pitié, l'Hôpital de la, account of . i 281

Pitt, Mr., and Hunter's museum . iv 86

Pitting in smallpox, to prevent xv 383, xxiii 307

Pitville spa, at Cheltenham . . iv 555

Place, great, quotation from Bacon on xxiv 598

Placenta, absorption of . . . vii 255

part of . . iii 142

adhesion of, Dr. Ramsbotham on xv 527

to the forehead . . viii 17

auscultation of . . . viii 371

case of calcareous state of . v 115

discovery of tufts of . . xi 540

double function of . . xv 273

Dr. Reid on structure of . xi 540

effusions of blood into . . x 86

eleven weeks' retention of . xiv 236

extraction of . . . xix 426

formation of . . viii 42, ix 24

Hamilton's mode of separating iii 141

human, structure of . . xx 299

incipient formation of . . xx 304

in different animals . . ix 26

in excessive liquor amnii . viii 566

management of, in labour . viii 46

most frequently on left side . viii 371

Mr. Dalrymple on . . . xv 148

organized blood in . . xx 533

paralysis of seat of . . . xv 105

partially detached, sounds in . viii 373

piping, hissing sound of . . viii 372

position of . . . . . x 285

presentation, partial, hemorrhage in ii 83

puncturing membranes through x 99

remark of Levret on site of . viii 373

retained, on ergot of rye in . ii 524

retention of a portion of . iii 141

Dr. Davis on . iii 409

in 16,434 labours . ii 73

remarks on . . ii 84

site of, Naegele on . . xv 316

structure of . . . ix 25, xi 459

termination of vessels in . ix 29

twin, mode of delivering a . iii 413

union of, with uterus . . x 90

yellowish-gray masses in . x 86

Placenta prævia, ballotement in . x 93

causes of . . . . x 89

diagnosis of . . . x 92

Dr. Simpson on . . xx 535

hemorrhage in . . . x 91

how hand to be passed in ii 83

in 16,434 labours . ii 72

lesions in . . . . x 87

signs of . . . . x 85

the plug in iii 525, xxiii 287, 289

treatment of . . xxiii 285

Placentæ of twins generally united ii 90

Placental and uterine circulation . xi 541

circulation . . . . . viii 44

hemorrhage, diagnosis of . x 92

*not* slow . . . . ii 81

murmur, auscultation of . i 90

Dr. Breventani on . . v 167

from pressure on iliacs . i 486

presentation, fatal case of . iv 374

souffle, mistake respecting . xii 489

sound, cause of . . . v 168

Placental sound, disquisition on . i 485

vessels, peculiarities of . . x 90

Plague, and cholera, contrasted . xvi 298

and quarantine, works on . xvi 289-90

Dr. Bowring on . viii 233

and the exanthems, different . i 375

at Alexandria, in 1834 . . xix 44

cases to prove it contagious . xv 152

centres of infection in . . xxiv 256

-clothes, Turkish traffic in . xvi 303

coexistent with syphilis . xix 46

commencement and cessation of xvi 298

communication of . . . xix 45

concentration of the poison of . xvi 300

conditions of its development . xxiv 247

contagion of . . . . xix 41

anecdotes of . . viii 233, 236

by fomites . . . xvi 302

by clothes, &c. . . xix 45

Clot Bey on . . . i 249

contagious nature of . . viii 317

contagiousness of . . viii 233, xii 93, xvi 290, 300

Dr. Bowring on . . . vi 582

Dr. Bulard on . . . v 560

Dr. Prus on . . . xxiv 242

Dr. Shapter on . . . xii 92

Dr. White on . . . xxiv 242

effect of civilization on . . xviii 252

effects of inoculation of . . xvi 291

endemic and epidemic nature of xvi 297

epidemic characters of . . xxiv 251

experiments on infection of . v 561

extraordinary case of, in Malta xvi 304

from a trunk, case of . . xvi 303

from clothes of victims of . xvi 303-5

Galen's account of the . . v 194

importation of . . . xvi 292

in Egypt, before Christ . . xxiv 246

causes of . . . xix 366

Clot Bey on . . . i 248

observations on . . xiii 94

in French and English armies xvi 294

in Malta, in 1813 . xvi 292, 294

and Corfu . . . ix 75

inoculation with matter of v 561, xii 93

in relation to epidemic foci . xxiv 254

in the Levant and Turkey . xvi 307

in the 'Zebra' and 'Castor' . xvi 299

latency of . . . . xvi 302-5

Lorinzer and Hecker on . . v 191

ludicrous precautions against . viii 236

means of destroying the . . xxiv 250

medical commission on . . xvi 306

morbid anatomy of . . viii 550

appearances in . . v 560

M. Segur-Dupeyron's case of . xxiv 258

not contagious . . vi 582, xiii 258

of the second century . . v 193

nature of . v 194

origin of . v 193

progress of . v 194

origin of . . . v 192, xiii 94

and prevalence of . . xvi 306

Plague, parliamentary correspondence on xix 41

report on . . . xvi 290

period of latency in . . xxiv 257

possibility of eradicating . xix 47

precautions against . . xxiv 259

prevention of . . . xvi 307

propagation of . . . xvi 291

proposed antidote for . . xix 49

question on propagation of . xxiv 253

questions by Government on . xix 43

real source of, in Egypt . . xxiv 248

recapitulation of review on . xxiv 242

remedies for . . . . v 562

report of French Academy on . xxiv 245

sea air unfavorable to . . xvi 300

spontaneous, observations on . xxiv 246

sporadic, cases of, in Egypt . xvi 297

non-contagiousness of xxiv 252, 256

susceptibility of persons to . xxiv 252

syphilis, mistaken for . . xxiv 258

transmissibility of . . . xxiv 253

treatment of . . . . . v 561

Plagues, effect of civilization on . xviii 248

of Russia in 1551 and 1656 . i 598, 601

Planets, on the music of the . . xviii 170

organic life in the . . . xix 163

Plants, and animals, analogy of . ix 495

analogies in . . vii 170

development of xix 165, 174

dispersion of . iii 367, 370

nutrition of . . iv 12

origin of . . iii 367, 370

similar growth of ix 495, 520

ascent of the sap in . . iv 21

assimilation of . . . iv 22

carbon by . . . xi 437

hydrogen by . . . xi 439

nitrogen by . . . xi 440

azote the principle of food in . xiii 487

capacities for variation in . xxiv 58

Carus on the metamorphosis of v 85

cellular, mode of nutrition of . iv 12

change of organs in . . iv 28

changes in the cells of . . ix 501

curious discovery relative to . iii 582

depositions in the cells of . ix 502

development of heat in . . iv 24

digestion of . . . . . v 382

diseases of . . . . . iv 19

dotted cells and ducts in . ix 503

effect of cultivation on . . xvii 25

sunlight on . . . iv 23

effects on soil by excretions of iv 27

elementary organ of . . ix 499

essential food of . . . iv 17

evolution of carbonic acid by . iv 23

heat by . . ix 545

excitability of . . . iv 30

excretions by the roots of . iv 27

food of . . . . xi 437, 441

formation of cells in . . ix 498

galvanic electricity in . . iv 10

generative process in . . ix 500

Plants, how nourished by humus . xi 439

hybrids in . . . . iv 29

impregnation in . . . iv 29

influence of nitrogen on . vi 552

inorganic constituents of . xi 441

instinctive actions of . . iv 30

intercellular substance of . ix 502

microscopical structure of . ix 495

morphology of . . . iv 2

no comparative anatomy of . v 86

nutritious secretions of . . iv 26

organic mucus of . . . ix 502

organization of . . . xx 153

organography of . . . iv 2

origin of tissues of, from gum ix 497

pabulum of, only two forms of iv 16

purification of the air by . iv 23

regeneration of . . . xvi 209

reproduction of . . . iv 28

reproductive process in . . iv 561

respiration of . . iv 22, 24, v 382

special secretions in . . iv 26

structure of the parts of . iv 28

supplied with ammonia by dung xi 441

varieties in . . . iv 29

vascular, alimentary system of iv 13

vessels of . . . . ix 504

vital action of . . . xi 437

Plasma, aplastic . . . . xvii 482

caco-plastic . . . . xvii 482

euplastic . . . . xvii 482

(liquor sanguinis), function of xv 278

nature and origin of . . xvi 213

or liquor sanguinis . . xiv 585

Plaster, adhesive, Mr. Liston's improved v 459

adhesive, of Hamburg codex . ii 255

Liston's adhesive . . . xiii 265

improvement on . . xiii 266

Plaster of Paris for club-foot . iii 514

Plastic operations, after-treatment in xxi 301

Dr. Mütter on . . xxii 381

forms of . . . vii 397

gangrene in . . . vii 395

indications for . . vii 393

of M. Dieffenbach . . xi 197

surgery, birthplace of . . vii 388

contraction in . . . vii 394

Dieffenbach on . . xxi 292

Dr. Zeis' manual of . . viii 384

erysipelas in . . . vii 396

general treatment in . vii 396

its use in cancer . . vii 412

loss of temperature in . vii 394

nomenclature of . . xii 388

observations on . . xix 396-9

progress of . . . vii 389

sensibility of part in . vii 395

treatises on . . vii 386, xix 396

Plate, and explanation of it . . xv 259, 280

Plates, anatomical, effects of, on students ii 197

Hindustani anatomical . . xxii 227

M. Rayer's, account of . . ii 157

of morbid anatomy, Dr. Carswell's i 152

Plates of the skin, explanation of ii 615
Platina, alterative virtues of xi 523
  chlorides of, poisons xi 523
  efficacy of, in syphilis xi 523
  employment of, in medicine xi 523
PLATO, his philosophy v 82
  metaphysical theories of xxiii 98
Plea of insanity, the twelve judges on the xvi 273
Pleasure and pain, feelings of, seat of xxii 512, 514
  Unzer on xxiv 197
Pleasures, selfish and sympathetic i 319
PLEISCHL, Dr., on the use of salicine i 570
Plethora anatomica iii 213
  arteriosa, or erythrosis iii 212
  hæmato-poietica iii 213
  nervosa, chiefly in females iii 213
  venosa, or cyanosis iii 213
  vera, two varieties of iii 213
Plethora, asthenic, nature of xvii 489
  treatment in xvii 489
  different forms of iii 212
  nature and kinds of xiii 331
  of the venous system xiv 330
  Prof. Neumann on iii 212
  state of the blood in xvii 138
  sthenic, hemorrhage in xvii 489
  nature of xvii 489
  symptoms mistaken for v 387
  use of arsenic in v 605
Pleura, cartilaginous bodies in iv 46
  exudation of, free cysts in xxiv 119
  false membrane of xv 87
  fibrous layer in the v 424
  membrane of v 220
  morbid anatomy of iv 43
  perforation of xv 92
Pleural scalenus muscle xix 104
  surfaces, signs of collision of ix 315
Pleurisy, after flogging ix 317
  chronic, treatment of xiii 364
  diagnosis of xx 247
  distension of chest by xix 112
  doubts on blisters in ix 317
  effusion in xxi 515
  from emphysema xix 112
  in children, frequency of xv 323
  in infants vii 82
  in American army xiii 430
  of diaphragm xxiv 178
  percussion in xix 112
  physical condition in xix 111
  remarks on treatment of xxi 511, 514
  secondary xix 111
  Valleix and Dunglison on xx 450
Pleuritic effusion, bronchophony in xiv 177
  case of, relieved xxii 561
  dilated chest in vii 367
  effusions, paracentesis in xii 250, xx 451
  lesions, peculiar to phthisis xvi 430
Pleuritis, chronic, effects of, cases of iv 479
  fatal, singular case of xvi 322
  frequency of, in phthisis xix 113
  scorbutic, at Kronstadt xii 250
Pleurodynia, diagnosis of vi 356
  French remedies for vi 356
  symptoms of vi 355
  treatment of vi 356
Pleximeter, its advantages in percussion iii 185
  of India rubber iii 187
Pleximeters, and percussion-hammers xiv 180
Plica Polonica, observations on xix 241
  pathology of xii 247
PLOUCQUET, his test xvi 309
  Devergie's remarks on ii 410
  of infant lungs vi 64
Plug, Dr. Hamilton's objections to the iv 406
  for holes in the palate xxi 307
  in the throat, infanticide by ii 414
  in uterine hemorrhage x 80, 98
  premature labour induced by xiii 249
  the best, for uterine hemorrhage iii 525
  use of, in placenta prævia iii 525
  value of, in uterine hemorrhage ii 82
Plugging, in uterine hemorrhage xiv 368
PLUMBE, Mr., on diseases of the skin iv 175
  on his forceps for porrigo ii 153
Plumbi acetas and opium, in cholera v 280
Plunge, on the hydropathic xxii 450
Plurality of birth, cases of xxiii 280
  more dangerous than single ii 90
  of children, Report on xx 529
Pluviers, sudden deaths at, in 1747 vii 69
Plympton lunatic asylum xix 324
Pneumatic medicine, remarks on xxiii 566
Pneumogastric, algor of xvii 414
  ardor of xvii 414
  hyperesthesiæ of xvii 414
  pruritus of xvii 414
Pneumogastric nerve, and branches v 309
  branches of viii 265, 269
  death from section of xi 519
  Dr. Reid on viii 265
  functions of xi 517
  Mr. Cock on vi 60
  nerves, influence of ix 489
  on tying iii 154
  offices of iii 155
  report on xxii 283
Pneumonia, action of emetic tartar in xx 81
  affections of bronchi in xv 557
  a first stage in iv 314
  after operations, &c. xviii 365
  albuminous induration in xviii 338
  all forms of, seat of xi 406
  anatomical stages of xix 110
  anatomy of products of xi 407
  and bronchitis, distinct iv 314
  and its consequences xviii 337
  aneurism mistaken for xi 516
  antimonial treatment in iv 317
  a peculiar lesion in xiii 386
  as to previous attacks of xv 561
  at different ages, table of xiii 388
  auscultatory signs in xiii 395
  blisters in xiii 410
  bloodletting in xiii 402

Pneumonia, Bouillaud's bleedings in . xiii 405-9

bronchial respiration in . . xiii 395

bronchophony in . . . xiii 396

not always in . . i 487

cardiac concretions in . . xiii 386

case of, saved from nature . xxii 560

catarrhal . . . . xv 89

causes of . . . . xv 559

change of position in . . iv 317

chronic . . . . . xiii 398

complications of . . xiii 391, 398

condition of the blood in . . xx 76

crepitant rhonchus in . . xiii 395

crepitation not always in . . i 487

crises in . . . . . xiii 397

diagnosis of . . . . xiii 401

diagnostic sign of . . . ix 489

dilatation of bronchi in . . xv 558

of chest in . . xiii 394

distension of chest by . . xix 112

double, not frequent . . xiii 384

Dr. Addison on . . iv 356, xx 226

Dr. Hughes on . . . xvi 320

Dr. Stokes on . . . iv 313

on diagnosis of . . iv 316

on treatment of . . iv 316

dyspnœa in . . . . xiii 393

effects of . . . . xviii 337

of bloodletting in i 405, iii 457, xx 80

elementary seat of . . . xiii 385

emetic tartar in . . . i 414

four varieties of . . . xv 88

fremitus in . . . . xiii 396

frequency of bleeding in . . xiii 405

frequency of, in the old . . ii 546

gangrene in . . . . xiii 398

gastritic complication of . . xiii 387

general bloodletting in . . iv 317

granular induration in . . xviii 338

gray induration in . . . xviii 338

homœopathic treatment of . xxii 581

incipient, diagnosis of . . iv 314

in deformed chests . . . xiii 389

infantile, blisters in . . xv 571

calomel in . . . xv 570

depletion in . . . xv 569

diagnosis of . . . xv 568

emphysema in . . xv 558

French writers on . . xv 543

general management in . xv 572

morbid anatomy of . . xv 544

mustard poultices in . xv 572

peculiarity of . . . vii 77

percussion in . . . xv 568

pericarditis in . . . xv 559

physical signs in . . xv 565

pleuritis in . . . xv 558

report on . . . xx 556

respiration in . . . xv 564

stimulants in . . . xv 571

symptoms of . . . xv 562

tartar emetic in . . xv 570

treatment of . . . xv 568

Pneumonia, infantile, tubercles in . xv 559

influence of age on . . . xv 560

of climate on . . . xiii 390

season on . . . xv 559

sex on . . xiii 388, xv 560

trades on . . . xiii 389

winds on . . . xiii 390

infrequency of, at sea . . xiii 389

in phthisis . . . xvi 430, 444

in children . . . xvii 310

in relation to catarrh . . xv 561, 564

in Sweden, Dr. Huss on . . xxii 468

interstitial . . . . xv 90

intervals in bleeding in . . xiii 405

in the American army . . xiii 430

Homœopathic hospital . xxiii 609

right and left lung . xiii 384

in typhoid fever, remarks on . ii 52

is bleeding useful in . . xiii 402

lectures on . . . . xi 516

lobular, in children . xii 354, xv 555

stages of . . . xv 556

morbid anatomy of . . xv 88

mortality in 304 cases of . . xiii 411

M. Grisolle on . . . xiii 382

no instant cure of . . . i 409

of children, Dr. Berton on . xv 322

Dr. West on . . xv 543

morbid anatomy of . xii 353

prognosis in . . xii 360

report on . . . xvii 559

sounds in . . . xii 358

statistics of . . . xv 543

treatment of . . xii 361

of infants, its character . . i 253

M. Bouchut on . . xx 362

peculiar . . . xi 207

treatises on . . . xii 350

of old age, signs of . . . xvii 113

symptoms of . . xvii 113

of the aged . . . . ii 543

is acute . . . xvii 113

medicines for . . xvii 113

treatment of . . xvii 113

of the old, causes of . . iv 502

changes by . . ii 543

diagnosis of . . ii 543

mode of attack of . iv 502

state of pleura in . ii 546

bronchi in . ii 546

symptoms of . . iv 503

on a sudden chill causing . xiii 391

on bleeding in all cases of . xiii 404

operation of nitre in . . xx 81

opium in, M. Louis on . . i 414

periods for bleeding in . . xiii 404

previous health in . . . xv 561

prognosis of . . . . xiii 401

prune-juice sputa in . . xvii 521

puerile respiration in . . xiii 396

pungent heat of surface in . iv 357, 358

relapses of, Dr. Lebert on . xxii 25

relative mortality from . . xxiii 610

Pneumonia, respiration in . . vii 263
results from bleeding in . . xiii 408
saignées coup sur coup in . xiii 405-9
scrofulous, mercury in . . ix 492
seat of . . . . . . xviii 337
in parenchyma . . iv 314
secondary . . . . . xv 561
semeiology of stages of . . ix 317
senilis, description of . . xvii 112
signs of, in still-born child . xxiii 298
site of pain in . . . xiii 393
Skoda's practice in . . xxiii 609
treatment of . . . xxii 591
sputa in . . . . . xiii 394
statistical researches on . . x 262
suppuration in . . . . xiii 398
symptoms of . . . . . iv 357
table relative to stages of . xiii 384
termination of . . . xiii 397
the rusty viscid sputa in . iv 316
tuberculous infiltration in . xix 111
treated without bleeding . xx 252
treatment of . . . ix 491, xiii 402
by tartar emetic . . xiii 409
typhoid, bronchophony absent in i 488
crepitation absent in . i 488
frequent in Dublin . . iv 316
no crepitation in . . i 488
treatment of . . . iv 317
two kinds of, in old persons . ii 545
very fatal in the aged . . i 407
vesicular . . . . . xv 557
in children . . . xii 354
rhonchi in . . . ix 317
with acute rheumatism . . xx 184
bronchitis . . .xiii 392, 399
delirium . . . xiii 400
tremens . . . iv 316
heart disease . . . xiii 399
jaundice . . . xiii 399
phthisis, signs of . . xiii 400
pleurisy . . . xiii 398
pneumothorax . . xiii 399
polypus of heart . . vi 547
without physical signs . iv 315, xix 111
Pneumonic abscess, Dr. Stokes on iv 315
diseases, on collapse in . . x 285
expectoration, Dr. Remak on xxiii 504, 506
structure of . . . xxiv 123
granulations, anatomy of . xv 89
phthisis, Dr. Addison on . xxiii 421
sputa, bronchial coagula in xxiii 505, 506
Pneumothorax, and empyema, case of ix 394
cause of sounds in . . . vii 569
from paracentesis . . . xiii 371
history of . . . . . xx 249
traumatic, case of . . . xii 552
Pnoeum, a nostrum sold by Hahnemann xxii 565
Pock, remark on the term . . vi 483
Pocket-books, medical, censure on vi 202
Poem on pathology by Dr. Bang . ii 301
Poets, Mr. Neville on the madness of iii 190
Poiseuille, M., his hemodynamometer viii 336
Poison-book kept in Russia . . iv 265
Poison by snakes, treatment in . xii 138
contagious, convertibility of . xxiii 317
disappearance of, from the body xx 324
definition of a . . . xvi 75
Dr. Alison on the action of . xviii 218
imbibition of, after death . xi 54
inclosed in a tube in a vein . iii 17
inert on vegetables . . ii 518
many unnoticed deaths from . xx 326
means of preventing . . i 596
musk as a, in dogs . . i 245
of serpents, remarks on the . viii 315
of snakes, Dr. Davy on . . xii 137
the toad, Dr. Davy on . xii 136
Poisons, absorption of . . . xvii 260
act through the circulation . i 559
action of . . xiv 199, ix 569, xx 321
on the muscles . . xvi 418
vegetables . . iii 17
through circulation . iii 17
nerves . . . iii 16
and remedies, on the action of xiv 520
antidotes to . . . . . xx 322
corrosive, action of . . ix 56
development of, in the body . xx 67
Dr. Christison's treatise on . xx 317
experiments of Addison and
Morgan on . . . iii 17
experiments of Segalas on . iii 17
general laws of . . . v 354
in the blood, expulsion of . xvii 486
irritant, report on . . . xviii 533
Liebig on the action of . . xi 442
mineral, Dr. Prater on . . xxii 545
mode of operation of . . viii 314
morbid, blood the seat of . xvii 485
Dr. Williams on . v 353, xiii 89
peculiarities of . . v 355
morbific, peculiarities of . xiii 89
narcotic, report on . . xviii 557
nature of morbid . . . v 354
physiological action of . . xx 317
their probable mode of action iii 18
to be found in the blood . xx 320
treated of in the Susruta . xxiii 543
various writers on action of . i 559
Poisonous cattle in America . . xviii 555
Poisoning, antidotal treatment of . xviii 553
by acetate of lead . . . iv 209
case of . . . xii 249
antimonial vapours . . x 580
arsenic, bleeding in . . xi 45
arsenic, cases of iv 359, 361, viii 568
arsenic, case of . . xiv 122, xvi 322
cases of, cured . . i 572-3
cured . . . . . vii 563
Dr. Von Speez on . . iv 237
in beans, case of . . iii 528
by carburetted hydrogen . ix 380
charcoal vapours . . ix 377
coal gas, cases of . ix 380, xv 252
copper . . . . . ix 58

Poisoning, by corrosive sublimate xvi 77, xviii 342

by decayed sausages . . xi 444

diseased mutton, case of . xviii 332

disulphate of quinine . . xviii 164

feather white wine . . xiv 307

hydrocyanic acid . . iv 533

liquor potassæ . . . iv 239

mercury, albumen in . . ix 57

gluten in . . ix 57

mineral acids, pylorus in . iii 540

remarks on . iii 540

minute dose of laudanum . xiii 574

mistake . . . . i 306

of chemical name . xviii 361

opium, case of, by Mr. Taylor xxiii 416

organic substances . . xi 444

oxide of zinc, case of . . vi 221

putrid pickled salmon . . xvii 25

seeds of stramonium . . xix 305

prussic acid, the shriek in . xxiii 423

rotten eggs, case of . . ix 267

sulphate of indigo . . vi 244

sulpho-cyanic acid . . viii 258

sulphuric acid . . . vi 245

cases of . iii 539, ix 391, xiv 584

remarks on . . xx 327

the rattlesnake . . . vii 578

cases simulating . . . ix 55

cured by counter-irritation . vii 62

detection of opium in . . ix 58

diagnosis in cases of . . xviii 534

effect of civilization on . . xviii 242

evidences of . . . . xx 323

French Academy on . . xiii 197

from decayed meat, cases of . xv 238

from yew berries . . . iii 564

infants, by opiates . . xviii 504, 558

in England, statistics of . . ix 278

France, statistics of . . i 596

Turkey, its frequency . . iv 389

jurisprudence of . . . xvi 75

marks of disease in . . xx 323

M. Orfila on . . . . xi 37

narcotic, undetected . . xviii 535

of a child by laudanum, case of xxiii 299

on compound . . . . ix 59

secretion of urine in relation to xxi 420

vegetables, experiments on . ii 518

warning, case of suspected . ix 268

Poke, juice of the, in asthma . . xix 25

Polarity, opposite, of the tissues . xxiv 133

Police, medical, observations on . xv 329

Political economy, Sir A. Carlisle on vi 187

medicine, Dr. Maunsell on . viii 547

importance of . . viii 547

Polli, Dr., on fibrine . . . xxi 542

Polyæmia or plethora, remarks on . iii 212

Polycephali, inoculation with . . xx 414

Polycephalus cænurus, account of . xx 407

Polycholia, Dr. Horaczek on . . xxii 312

Polydesmus maculatus, nervous system of xx 494

Polygala senega, a valuable medicine iii 510

Polygastric animalcules, Mr. Addison on xv 274

Polynesian islands, on the peopling of xxiv 472

Polynesians of the Pacific . . xxiv 468

Polype, on voluntary motions of the v 489

Polypi, and fibrous tumours of uterus iv 371

and polypoid tumours of uterus xxiv 510

Dr. Gross on . . . . xxiii 68

fibrous, of uterus, treatment of xiv 558

nasal, formation of . . xxiv 121

new instruments for tying . viii 237

mode of tying . . vii 253

obstruction in labour from . xi 502

of the heart, causes of . . ii 341

M. Bouillaud on . ii 340

M. Legroux on . ii 339

nature and effects of ii 341

pus in . . . ii 340

prognosis of . . ii 341

symptoms of . . ii 341

of the rectum in children . xvii 561

uterus, frequency of . xviii 7

ligature of . . xviii 14

site of . . . xviii 7

varieties of . . xviii 5

vagina, Lisfranc on . xviii 17

vulva, Lisfranc on . xviii 17

soft, of the uterus . . . xxiv 510

uterine, absence of vascularity in iii 403

avulsion of . . . xviii 13

caution in removing . iii 403

complications of . . xviii 11

enucleation of . . xviii 13

excision of . . . iv 183

hemorrhage after excision of xviii 16

from . . . xiii 543

improved canula for . xix 354

in old maids . . . iv 372

ligature of . . . iv 184

ligatures on . . . iii 403

on dividing the peduncle of xviii 15

palliative treatment of . xviii 10

period for removing . xviii 12

remarks on removal of . xviii 11

statistical table of . . iv 370

Wenzel's plates of . . iii 403

Polypous concretions in the heart . xiii 387

excrescences in the bladder . xi 361

on uterine . . . iv 183

Polypus, and inversion, of uterus, diagnosis of . . . xviii 8

aperture at lower part of . vi 93

bronchial . . . . vi 268

expectoration of . . ii 554

case of uterine, cured . . viii 238

elastic, from epiglottis . . iii 72

fibrous, on the lip of the uterus xxiii 281

inflammation in . . . vi 93

mistaken for prolapsus uteri . xx 138

of frontal sinus, case of . . xxi 186

of heart, before death, case of vi 547

case of . . . ii 340

of the uterus, bleeding from . iii 136

case of . . . xiv 583

circular depression on xviii 9

Polypus of uterus, cure of, by ligature iii 137

diagnosis of iii 136, vii 451, xvii 509

Dr. Hamilton on . iii 135

Dr. Lee on . . ii 165

Dr. Lever on . . xvii 508

excision of . . iii 137

fibrous . . . xviii 344

alterations of . xviii 6

envelope of . xviii 5

pedunculation of xviii 7

states of . . xviii 5

hemorrhage from . xvii 509

ligature of whipcord for xvii 510

mistaken case of . iii 136

natural cure of . iii 137

prognosis in . . iii 136

riband-like . . xviii 8

spongy . . xviii 344

spontaneous cure of xviii 10

strictly cellular . xviii 345

structure of . . iii 135

symptoms of . . iii 135

treatment of . . xvii 510

suffocation from elastic . . iii 72

true, of the auricle . . xx 223

uterine, Dr. Davis on . . iii 403

hemorrhage in . . vi 93

reduction of . . . xxi 86

safety of excision in . xv 528

sensibility of . . . xxi 88

von Graefe's instrument for . vi 94

Polypus uteri, complicating labour . xx 531

diagnosis of . . . vi 93

from cancer . . vi 94

Dr. Oldham's summary on xviii 345

hemorrhage from . . iv 370

ligature for . . . iv 370

new knife for . . xxiii 295

pain from tying . . iv 370

preference of knife for . iv 369

report on . . xx 543, xxiii 293

spontaneous cure of . xvii 546

various kinds of . . xviii 344

Polysarcia cured by iodine . . v 164

Polystoma sanguicola, described . v 555

in human blood . v 555

Pomegranate-root-bark, decoctions of iv 113

Pomegranate, *root*-bark only, for tænia iv 112

Pommade ammoniacale of M. Gondret vii 65, 57

épispastique au Garou . . xiv 441

Pompholyx, varieties of pemphigus xv 385

Pongo and ourang outang, identity of x 251

Pontine marshes, malaria of the . i 351

Poor, the, deficient food of, evil effects of ii 384

effect of civilization on xviii 242, 245

evils of, and their remedies . xix 135

health of, Mr. Chadwick on . xv 328

in Denmark, charities for . iii 572

Edinburgh, destitution of . xi 25

Glasgow, destitution of . xi 26

Scotland, Dr. Alison on . ix 544

legal relief to . . xi 27

Poor, in Scotland, management of xi 1

wretched state of . xi 26

labouring, Dr. Combe on their food ii 384

management of, in Denmark . iii 572

nutritious and cheap soup for xiii 496

on medical aid to . . . xix 229

relief to the sick . . iv 557

modes of relief to . . xix 132

physicians for . . . xiv 407

wholesome houses for . xix 134

Poor-law, medical relief under . v 310

new, medical relief under vi 586

-laws, paupers, &c., observations on xxii 348

man's path, stumbling-blocks in xix 139

-rates, costliness of the . . xv 340

Pope Sextus V, anecdote of . xxiii 162

Popliteal aneurism . . . xxiv 421

case of . . . vii 153

relapsed . . vi 67

cured by pressure . ix 277

artery, fusiform figure of . vi 45

Poppy enemata, in typhus fever . v 361-62

Popular advice, rather hazardous . v 391

medical books, remarks on . vii 513

names of diseases, remarks on iii 446

riots, observations on . . xv 339

Population, and subsistence . . xvi 229

civilization checks . . . xv 339

climate in relation to . . viii 40

diseases from density of . . xi 431

effects of civilization on . xviii 237

increase of . . . . . xiv 450

moral checks to . . . xvi 229

principles of, remarks on . xv 337

true moral checks on . . xiv 451

vice and misery increase . xv 337

Pork, deleterious effects of . . iii 272

Porosity, and imbibition, Magendie on viii 311

in relation to fomites . . i 378

Porrigo, Alibert on the treatment of i 578

a linen cap preferred in . . iii 143

anatomy of the parts in . . vi 429

benefit of leeches in . . iv 179

contagious nature of . . vi 430

Dr. Dick's classification of . vi 429

Dr. Roots on . . . . iii 143

Dr. Williams's pathology of . xiii 97

irritants in . . . . . vi 431

M. Rayer on . . . . ii 152

of vegetable origin . . xx 267

on plucking out the hair in . vi 430

Plumbe's forceps for . . ii 153

successful treatment of . . xiii 97

treatment of . . . . vi 430

in Paris . . . . . i 578

various remedies in . . iii 143

vegetable parasites in . . xiii 485

Porrigo decalvans, nature of . . vi 430

lupinosa, a preparation of . vi 430

microscopy of . . . xxiii 508

seat of . . . . . vi 429

Porta, Prof., on ligature of arteries xxii 89

Portal blood, composition of . . xvi 214

Portal circulation, physiology of . xiii 262
system of the kidneys . . xiv 567
Porte-ligature pharyngien . . vii 253
Porter, Mr., on aneurism . . xii 438
on the larynx and trachea . v 423, 427
Portio dura, Bellingeri on the . ix 143
of auditory nerve, remarks on v 283
paralysis of the . . xiii 553
question on neuralgia of the xiii 148
Portrait Gallery, Medical . vi 194, ix 543
Mr. Pettigrew's . vii 225
Portugal, practice of medicine in . vi 285
present state of medicine in . vi 283
surgery in . . . . . vi 286
the medical press in . . vi 286
Position, effects of, in acute diseases xi 59
in labour, importance of . viii 46
Positive nosology and medicine . xxiii 37
Posology, homœopathic . . xxi 229
Posseting of infants . . . xx 238
Posthioplastic, etymology of . . vii 412
operation . . . . . vii 412
Postscript to the Review . . . xxiv 585
Potash and soda, modes of obtaining xvi 353
bichromate of, poisoning by . xviii 552
curious transformation by . viii 359
sulphate of, poisoning by . xviii 556
Potassæ liquor, its use in phthisis . xiv 196
poisoning by . . . iv 239
remedial uses of . . viii 359
Potassium, as an element of food . xvii 4
Potato, constituents of the . . xvii 20, 29
-diet of the Irish peasantry . xvii 29
-disease, observations on . xxiii 582
-plant, Mr. Smee on the . . xxiii 581
Potatoes, nutritive qualities of . xiii 487
Pot-bellies, pitiful tenderness for . xiv 215
Potentia and power, on the terms . xii 385
Pott's disease, M. Richet on . i 574
Potter, Mr., on amputations . xiv 60
Poudrette, manufacture of, in France xi 17
manufacturers, health of . xi 17
Poultices, on the application of . xv 37
Mr. Josse's objections to . iii 118
Mr. Liston's objections to . v 464
substitute for . . . v 459, 464
Poverty, a cause of fever in Glasgow xviii 505
Powdered alum, in angina . . xiv 228
Powders, mode of inhaling . . xviii 296
observations on . . . . xvi 203
Powell, Mr., on the inductive philosophy . . . . . v 548
Power resigned reluctantly . . ix 163
Pow-wow Club, account of the . xvi 134
Practical men, remarks on . . vii 489
medical men, on mere . . iv 84
Practice, and experience, remarks on xxiii 157
and science, feud between . xvii 472
English medical, Dr. Marx on xv 29
French medical, state of . i 495
medical, on subdivision of . x 200
of medicine, learning the . xvii 122
reformation in . . xxi 262
Practice, of physic, works on the . ix 461
radical, radical fault of . . xxi 516
routine or mechanical . . xxii 121
simplicity in . . . . . xxiii 469
Practitioners, Dr. Kilgour on young i 359
examination of . . . . xix 199
keeping medicines, on . . xxi 525
medical, privileges of . . xvii 123
protection of . . . . xix 551
portraits of various . . xxii 121
registration of licensed . . xix 550
medical, responsibility of . i 306
responsibilities of the . . xxiii 244
scientific and empirical . . xiii 472
unlicensed . . . . . xix 224
Precordial elevation . . . vii 366, 368
Præcordia, prominence of, by palpitations i 448
Prague, lying-in hospital at . . xiii 109
Prater, Dr., on mineral poisons . xxii 545
Pratt, Mrs., and the Archbishop of Canterbury . . . vii 312
Praxagoras, the tutor of Herophilus xv 107
Praxis medica of Dr. Ludvig Bang ii 297
Prayer, on inability for . . . xv 427, 429
Precocious development in children viii 19
Precocity, mental, a sign of disease iv 61
Prediction, the power of, in science xx 141
Predisponents to disease, Mr. Travers on ii 6
Predisposition to disease . . xxiv 301
abnormal . . . xxiv 303
normal . . . . xxiv 302
to scrofula, on the . . . xxii 144
Prefatory notice, by the Editor . xxi 501
Pregnancy and childbed, diseases of x 544
areola of nipple as a sign of i 100, iii 405, iv 452
auscultation in . . iv 457, xiv 208
writers on . . . . iii 406
auscultatory signs of . . v 167
bursting of a varix in . . xvii 534
axiom for a woman during . xix 62
beneficial effects of . . iv 450
blood not always buffy in . iv 459
bluish tinge of vagina in . iv 458
vagina in . . . xiv 209
case of extra-uterine . . ii 570
supposed . . . viii 371
ventro-uterine . . xiii 507
cases of, in early age . . iv 459
consciousness of . . . vii 130
diagnosis of, by Dr. Hohl . i 107
from urine . . . xii 326
difficulties of diagnosis of . iv 451
diseases complicating . . viii 37, 50
disorders of, report on . xxiii 277
Dr. Hamilton on the signs of. iii 140
Dr. Martin on . . . viii 37, 50
Dr. Montgomery on . . xiv 207
Dr. Osiander on signs of . iii 247
duration of . ii 403-4, iv 463, vii 136
dyspnœa in . . . . . xxi 90
effects of imagination in . . iv 450
enlargement of abdomen in . iv 454

Pregnancy, examination in, Rœderer on i 108
extra-uterine, cases of iii 245, 522, vi 252, vii 257, xxi 419
Dr. Heim on . . . iv 493
gastrotomy in . . . xiii 545
in the sixth year . . ii 571
in 70th year . . . xiv 560
report on . . xvii 534, xx 527
sounds in . . . viii 370
symptoms of . . . iv 493
ten kinds of . . . v 254
the mother in . . v 255
fatal vomiting during . . xx 526
fermented liquors in . . xxiv 543
first, diagnosis of . . . i 109
gravidine in urine in . . xiii 571
hemorrhage in early . . xii 118
of the Fallopian tube . . vii 562
kiestein as a sign of . . xiii 568
a test of . . . . . xii 566
menstruation in, not rare . iv 451
mental depression in . . iv 450
milky, turbid urine in . . iv 459
M. Andral on the blood in . xvii 148
M. Jacquemin's test of . . iv 68
moral treatment in . . iv 449
movements of child in . . iv 454
Nauche's metroscope for . iv 458
nervous irritability in . . iv 450
new diagnostic sign of . . ii 274
os and cervix uteri in . . iv 455
on examining abdomen in . iv 455
fixing the term of by law . vii 137
propriety of bleeding in . . xiii 404
protracted, disquisition on . ii 404
psychological changes from . vii 132, 133
recent literature on . . xvii 533
recognised by the urine . . xii 539, 566
regular menstruation in . . iv 451
repletion during . . . iv 449
Rœderer on the areola in . . iv 452
signs and symptoms of . . iv 448
signs of . . . . . . xx 525
Dr. Kilian on . . xiv 208
Prof. Devergie on . . ii 403
report on . xvii 533, xxiii 277
spurious, remarks on . . iv 460
state of system during . . iv 449
umbilicus in . . iv 455
streaks in breasts during . . iv 465
suppression of menses in . . iv 451
treatment of diseases in . . iii 141
tubal, case of, with no signs . iii 245
ending in rupture . . iii 245
two auscultatory signs of . . v 596
ulcers of cervix uteri in . . xvii 534
uncertainty of signs of . . vii 131
use of the stethoscope in . . v 597
vaginal pulse a sign of . . iii 248
watery discharge in . . xvii 534
Pregnant single women, espionage of vii 131
women, fine paintings before . ii 367
longings in . . . viii 17
Prejudice, Bacon on . . . xxi 538
Prejudices, effect of civilization on . xviii 244
Premature labour, mode of inducing iv 404
on inducing . . iv 411
on the term . . vi 81
Pre-ordination in mesmerism . . xix 468
Prepuce, method of restoring . . vii 412
plastic operation on . . xxi 313
Presbyopia, lenses proper for . . xvi 362
power of vision in . . . xviii 422
Prescriber's Pharmacopœia . . xix 246
Prescribers, elegant, remark on . xxi 264
Prescribing, for names of diseases . ii 474
theory and art of . . . xvi 197
Prescriptions, four constituent parts of xvi 203
medical, perplexities of . . xxii 443
observations on . . . xvi 202
registration of, in Russia . . i 602
remuneration for . . . x 218
Presentation, kinds of, in labour . ii 78
of arm, case of . . . xiv 582
three times . . . xx 533
of head, in labour . . . xix 66
Presentations, Hohl on diagnosis of . i 114
in labour . . . . viii 46
analysis of . . . xiii 570
natural, divisions of . . xix 66
of face to the groin . . xii 472
Press, venal non-medical, evil of a . xix 234
Pressmen and compositors, phthisis in xviii 499
Pressure, after delivery . . . vi 567
atmospheric, influence of . . xxiv 98
cure of aneurism by . xix 525, xx 438
in aneurism, advantages of . xx 438
in venereal phagedena . . vi 564
on mortification from . . xxii 171
treatment of aneurism by xxi 490, xxii 373
vascular, within the cranium . xxii 413
Presumption in practice described . xxii 122
Preternatural labour, Dr. Hamilton on iv 405
report on . . xxiii 280
visitations to man . . . xix 472
Prevision, mesmeric internal . xix 449, 468
Priapism in spinal injuries . . iv 145
PRICHARD, Dr., and his work, eulogy of iii 376
his History of Man . . xiii 521
Natural History of Man xv 180, 183, xxiv 441, 482
Physical History of Mankind xxiv 441
retrospective address . . iii 160
works, estimate of . . xxiv 481
on insanity . . . . vii 1, 5, xvi 81, 87
man, object of the work . xv 180
mankind . . . . v 543, xii 519
the natural history of man . xxiv 49
the physical history of man iii 365
the physical history of mankind xxiv 49
treatment of diseases of the brain ii 596
PRICHARD, Mr., on hysteria . . vi 512
Prickly heat, description of . . xv 392
PRIESSNITZ, Vincent, account of . xiv 426
description of . . . xxii 436
diseases treated by . . . xiv 431

PRIESSNITZ, estimate of . . xiv 437

his channel of cure . . . xiv 428

cold-water practice . . xii 189, 192

houses for patients . . xiv 429

many patients of rank . xiv 427

mode of practice . . xxii 437

plan and theory . . xiv 428

process with cold water . xiv 429

regimen for patients . xiv 430, 436

three kinds of remedies . xiv 433-6

remark on the system of . . xvii 53

Priest-physicians of the Greeks xvii 456, 458

Primary existences and relations . xii 3

tissues, Mr. Slack's paper on . iv 6

Primitive trace in the embryo . . xv 267

Primogeniture in twin-cases . . ix 42

Primordial utricle in plants . . xxiv 224

Principle, true meaning of the word v 101

Principles, active, and simple ideas . xxiv 226

false, mischief of, in medicine . ii 4

of medicine, Dr. Williams's . xviii 91

Printers, diseases of, M. Chevallier on i 282

health of, questions and answers on i 283

longevity of . . . . . i 284

rules for the health of . . i 285

Printers' Pension Society recommended i 285

Printing in gold, poisonous . . vii 291

PRINZ, Dr., on regeneration of vaccine ix 76, 78

Prismatic cellular substance in shells xxi 77

Prisoners, Dr. Holst on the health of xii 498

in Norway, health of . . xvii 91

on the weight of . . . xxi 278

Prison at Auburn (U. S.), system in xii 500

in Philadelphia, system in . xii 500

life, frequency of phthisis in . xx 103

scrofula, causes of . . . xxiv 138

systems, American, insanity from xii 501

Prisons, dietaries for . . . xvii 49

effect of civilization on . . xviii 243

in Norway, Prof. Holst on . xvii 88

insane-wards in . . . xxiv 236

insanity in . . . . . xii 502

military, diet in . . . xxiii 168

mortality in, Dr. Baly on . xxiii 411

relative mortality in . . xii 499

PRITCHETT, Dr., on the African fever xvi 505

Privation, a cause of fever . . xi 28

Privet moth, nervous system of the . iii 291

Privies, want of, in towns . . xxi 344

Privileges, and protection, medical . xix 222

equality of medical, bill on . xix 550

of medical men, equality of . xix 202

Prize essays for 1837-8 . . . iii 287

medal, botanical . . . iii 293

medals of the Royal Society . iii 291

Proceedings of the Royal Society . ii 213

Processus vaginalis, in the female . xxii 288

Problems, medical, Messrs. Griffin's xxi 92

PROCHASKA, his anticipations of

Dr. M. Hall's discoveries . xxiii 202

on the functions of the common sensory . . . . . xxiii 205

on the reflex function . . v 623

PROCHASKA, Unzer the teacher of xxiv 210

views of, on nervous system . ix 113

Procreative instinct, seat of . . iv 425

PROCTER, Dr., on sympathetic nerve xviii 528

Products, inflammatory, of tissues . xxiv 118

morbid, pathology of . . xxii 329

Profession, new organization of the . xxi 531

on the choice of a . . . x 176

report on interests of the . . viii 607

Professions, effect of, on health . x 373

influence of, on longevity . i 194

morbility and mortality in . x 376

Professional emolument, remarks on xvii 192

persons, attendance on . . x 217

Professors, medical, in Prussia, number of i 296

Profluviæ, Dr. Bene on . . . i 14

Profunda femoris, varied origin of . xix 503

Progeny, effect of parental habits on vii 378, 383

effects on, in coition . . vii 378

Prognosis, Dr. Watson on . . xvii 124

French inattention to . . vi 131

in diagnosis . . . . xx 309

of Hippocrates . . . xvii 469

remark on . . . . . xvii 500

unfavorable, danger of . . xxiii 546

Prognostics of sex before birth . i 101

Progressive development . vii 178

evolution of vegetables . . vii 183

Prolapsus ani, new operation for . x 568

on the term . . v 484

Prolapsus of cord, causes of . . xv 315

mortality from . . xv 317

treatment of . . xv 319

of funis, Dr. Collins on . . ii 90

Dr. Denman on . . ii 91

Dr. Michaelis on . . ii 90

management of . . xiv 559

of umbilical cord . . . xv 314

uterus, and bladder . . xiii 563

Dr. Hamilton on . . iii 132

Lisfranc on . . . xviii 23

Prolapsus uteri, et vaginæ, cure of . iii 512

bound sponge for . iii 425

cause of . . . xxi 86

diagnosis of . . iii 132

Dr. Churchill on . . vi 96

Dr. Davis's operation for iii 404

during labour . . iii 425

exercise in . . . iii 135

Hamilton's bandage for iii 134

injections in, decried . iii 133

instrument for . . iii 404

irregular progress of . iii 132

new mode of treating . xi 197

new treatment of . viii 596

not from the ligaments iii 133

observations on . . xxi 176

operations for . xiii 564, xxi 326

Osiander's bags for . iii 134

pessaries in . iii 134, 425

Prof. Kilian on . . iii 424

pulled away by bits . vi 237

remarkable case of . vi 235

Prolapsus uteri, remarks on . iv 182, viii 45

report on . . . xx 540

ring for . . . xiii 549

treatment of . iii 133, vii 413

use of leeches in . . vi 96

pessaries in . xix 356

walking exercise in . iv 183

vaginæ, case of . . . xxi 87

remarks on . . . vi 90

vesicæ, remarkable case of . xxii 319

Proleptics, contributions to . . xviii 165

Proligerous disc in the Graafian vesicle i 611

Propensities, and emotions, nature of xxii 513

and feelings . . . . . xxiv 226

observations on the . . v 504

Property, definition of the term . v 324

remarks on the term . . xi 386

rights of, remarks on . . xix 134, 139

true meaning of the word . v 101

Prophetess of Prevorst, account of . vii 339

Prophylactic treatment, surgical . xiv 8

Prost, on continued fevers . . xii 296

Prostate, the adult, dimensions of . x 370

chronic enlargement of . . xiv 163

dilatability of . . . . x 370

enlarged, dysury from . . xiv 164

incision of, in lithotomy . . x 369, 371

knife best for dividing . . x 372

laceration of, in lithotomy . x 371

stricture near . . . . vi 166

Prostate catheter, form and length of v 467

gland, advice to the student on vi 165

applying substances to . x 529

cancer of . . . . xxii 306

detection of disease of . ix 260

diseases of . . . . x 528

perforating the . . . x 530

puncturing the . . . x 530

Prostatic secretion in the urine . xi 337

Prostitutes, change of voice in . iv 68

embonpoint of . . . . iv 68

infecundity of . . . . iv 69

in London, number of . . iv 64

in Paris, insanity in . . iv 70

number of . . . . iv 64

registration of . . iv 64-71

inspection of, in Paris . . iv 73

maladies of . . . . . iv 69

manners and habits of . . iv 66

menstruation of . . . . iv 69

number of, in Britain . . xvi 229

Parisian, ranks and classes of . iv 67

syphilis in . . . . iv 74

sanitary regulations for . . iv 72

sexual organs of . . . . iv 68

Prostitution, in London, Dr. Ryan on vii 539

in Paris . . . . . iv 63

-licences in Paris . . . iv 71

Parisian, causes of . . . iv 65

statistics of . . . . iv 65

Prostration, after operations, cases of xxiii 560

Mr. Travers on . . . . xxiii 559

on operations during . . x 587

Protection of medical practitioners . xix 222-31

Protective process, not inflammatory vii 426

Proteinaceous principle of aliment . xvii 10

Protein, change of, into gelatine . xiv 515

chemical alterations of . . xiv 513

production of . . . . xiv 511

the basis of vegetable albumen xiv 307

Proteine compounds in the body xix 249, xxi 541

Protococcus nivalis, or red snow vii 176, 182

reproduction in . vii 182

Proto-iodide of mercury, in syphilides xxiii 366, 370

ointment of . xxiii 370

pills of . . xxiii 368

Proto-ioduret of iron, new formulæ for xiii 550

Proto-nitrate, liquid, of mercury, use of xv 36, 37, 47

Protruded intestine, reduction of . xxiii 8

Prout, Dr., a contradiction of . xi 343

criticisms on, excuse for . . xvi 480

his Bridgewater Treatise . . xxi 109

doctrine of organic agents . xxi 120-3

peculiar views . . xvi 479, 485

on carbon in the blood . . iv 25

pulmonary exhalata . . i 242

stomach and renal diseases xvi 477, 486

urinary diseases, &c. . . xi 330

errors of . . xi 363

watery vapour from the lungs i 242

startling notions of . . . xi 333, 349

Proventriculus, of a pigeon, view of . ix 568

of birds . . . . . . ix 568

Provincial Association, Transactions of i 529, iii 159, iv 477, vi 1, 380, ix 216, x 461, xii 231, xiv 396, xx 508, xxii 319

Provincial Medical Association ii 300, 601, iii 290, iv 279, 551, 560, vi 287, 569, viii 303, 603, x 293

Eastern Branch ii 300

Southern Branch ii 606

Provisions, price of, effect of, on health xviii 155

Proximate principles, on knowledge of iii 56

Prudence and morality . . . xxiv 227

Prune-juice sputa in pneumonia . xvii 521

Prune-stone in air-passages, extracted xi 530

Prurigo podicis, in old age . . xvii 118

Prurigo, M. Rayer on . . . ii 154

Prurigo senilis, obstinate nature of . xv 392

ointments for . . xv 393

treatment of . . . xv 393

use of creosote in . . xv 394

urodialytic, of old age . . xvii 117

vulvæ, in old age . . . xvii 118

lotion for . . . xxi 87

Pruritus scroti, cured by lemon-juice xii 548

Prus, Dr., his work on irritation, &c. referred to . . . . . ii 5

on meningeal apoplexy . . xxi 409

plague and quarantine . xxiv 242

pulmonary emphysema . xviii 162

spinal pathology . . xii 535

Prussia, academy of naturalists in . ii 578

account of the hospitals of . i 303

Prussia, examen rigorosum of physicians in i 296
Frederick-William's medico-chirurgical institution in . . i 297
longevity and mortality in . viii 575
medical board of examination in i 301
civil functionaries of . . i 302
doctorate in, requisites for i 296
men of, their classification i 300
profession in . . . i 301
societies in . . . ii 577
medico-chirurgical academy for the army in . . . i 298
schools in . . i 298
medico-philosophical examination in i 296
military medical officers of . i 302
students in . i 297
number of medical lecturers in i 296
professors in . i 296
students in . i 296
physicians of, their examination i 300
their testimonium maturitatis i 296
present state of medicine in . i 289
provincial medico-chirurgical schools in . . . . . i 299
qualification of dentists in . i 608
surgeons of, their examinations i 301
tentamen medicum, in universities of i 296
vaccination in . . . i 609
Prussian army, revaccination of, 1834 ii 280
revaccination in . viii 261, xiv 237
colleges, annual school-fees in . i 296
universities, account of . . i 289
Prussiate of iron, its use in ague . ii 178
Prussic acid, antidotes for, remarks on xviii 554
case of suicide by . viii 258, 261
detection of . . xx 337
ink as an antidote for . xviii 553
its effect on snakes . . xii 138
mode of action of . . xx 318-20
new antidote for . . xviii 553
on poisoning by . . xx 333-38
power of action after taking much . . . . xviii 559
shriek in poisoning by xx 325, xxiii 423
silver as a trial-test of . xxiii 424
sulphate of iron as an antidote for . . . xviii 553
trials for poisoning by xviii 559, 560
Pseudarthrosis, treatment of . . xii 257
Pseudo-formations, melanotic . i 135
-organic productions . . xxiii 58
-plastic processes, Dr. Zimmerman on xx 71
-second-edition artifice . . xvii 531
-syphilitic affections . . v 8
-tissues, origin of . . . xxi 422
Psoæ muscles, on the actions of . v 140
Psora, the cause of chronic diseases xxi 232
Psorentery of MM. Serres and Nonat i 237
Psoriasis, and lepra, Mr. Wilson on xv 395
treatment of . xv 395
inveterata, aqua picea in . xxiv 48
syphilitic . . . . xxiii 361
universalis alba, case of . . iv 229
Psychal activity, general, opinion on xxiv 133
Psychical actions, distinction of . xxiv 196
endowments of animals . . xi 94
nature of man . . . xxiv 226
phenomena, substrata of . xix 308
spasms . . . . . . xvii 422
state in etherization . xxiii 553, 575
Psychico-physiological aphorisms, by Unzer . . . . xxiv 208
Psychological symptoms of idiotcy xxiv 2
Psychology of Van Helmont . . xvi 411
Psydracium, description of . . xv 117
Psuchrolousia, by Sir John Floyer . xxii 432
Pterygium, on the triangular form of xvii 234
*Ptolemies*, and Alexandrian school xxiii 93
Ptosis, Mr. Jeaffreson on . . xviii 413
traumatic, operation in . . x 26
Ptyalin, Dr. Wright's mode of obtaining xxiii 130
production of . . i 552
Ptyalism, mercurial, composition of viii 346
in pericarditis . . xxiv 565
in syphilis . . v 22, 23
Puberty, among Hindoo women . xxii 289
epoch of . . . . xxi 84
female, observations on . . v 408
in girls, age of . . . xvii 275
in relation to latitude . . xvii 276
insane acts at . . . vii 130
in women, remarks on . . xiv 574
moral effects of . . . vii 129
period of . . . . . xix 84
Public, advantage of writing for the vii 418
Publications in parts, animadversions on xx 120
Public-house disease, etiology of the v 133
fatality of the . v 132
Püchelt, Dr., on intestinal diseases xviii 310
on pelvic tumours . . . xi 500
on the venous system . . xx 430
Puel, M., his Military Medical Manual xix 380
Puerile respiration . . . ix 311
cause of . . . i 481
Puerperal case, from Gooch . . vii 498
contagion, Dr. Helm on . xiii 103
convulsions, Dr. Lever on . . xviii 341
emetic tartar in . ii 87, iv 410
neglected cases of . . ii 87
Prof. Romberg on . . xvii 422
rare afterward . . ii 87
rarity of . . . xvii 423
report on . xvii 539, xxiii 289
treatment of . iv 406, 408, xvii 422
disease, report on . . . xvii 540
rigors in . . . xiii 106
diseases, Dr. Helm's list of . xiii 104
treatises on . . . xiii 100
erysipelas . . . . xiii 120
fever, a blood-disease . . xxi 276
abscesses of vagina in . xiii 110
alkalies in . . . ii 489
a peculiar virus in . . ii 485
bleeding in . . . ii 488
bloodletting in . . ii 92
bluish-red tumours in . xiii 120

Puerperal fever—case of peculiar . ii 95

cases treated by ice . iv 518

change in type of . . xxi 276

chlorine in . . . ii 488

commencing . . . xiii 105

contagious nature of . ii 488

deposits of pus in . . ii 486

dissection after . . ii 483

Dr. Alexander on . . ii 490

his definition of . ii 491

Dr. Armstrong on . . i 39

Dr. Douglas on . . ii 482

Dr. Fergusson on . vii 482, 500

Dr. Gruber on . . vi 539

Dr. Lee on its origin . ii 484

Dr. Litzmann on . . xxi 275

Dr. Meigs on . viii 49, xxi 92

Dr. Michaelis on . . iv 517

dry air and ventilation in ii 488

epidemic causes of . . xiii 109

eruptions in . . . xiii 120

existence of, denied . xiii 101

exudations in . . xiii 114

fault of authors on . . ii 481

fetid discharge in . . ii 485

fomentations bad in . ii 93

from contagion . xiii 102, 111

from morbid blood . . ii 483

importance of discrimination in ii 490

infection of, from practitioners xiii 111

in Dublin, symptoms of . ii 92

Paris, 1840 . . xii 536

Prague, lesions in . xiii 114

its connexion with erysipelas ii 487

leeches in . . . ii 488

to labia in . . viii 56

linseed poultice in . . ii 93

mercurial influence in . ii 92

mercury in . . . ii 489

morbid anatomy of . xii 536

changes in . . xiii 121

Mr. Moore on . . ii 481

its origin . . ii 484

nature of . . . ii 482

not from phlebitis . . ii 485

inflammation . ii 484, 491

organs attacked in . . xiii 117

origin of . . . xiii 110

pathology of . . . iii 525

peculiar effects of . . ii 486

pneumonia in . . xiii 117

precaution against . . i 11

precautions against, by Dr. Collins ii 94

purgatives in . . . ii 489

real, discriminated . . ii 482

remarkable cause of . xxii 197

remarks on . . . viii 52

report on xvii 540, xx 536, xxiii 290

serious mistakes in . ii 491

singular origin of . . ix 571

sporadic origin of . . xiii 102

state of the blood in . ii 483-4

symptoms of the real . ii 482

Puerperal fever—syringing vagina in ii 93

treatment of . . . ii 92

use of ice in . . . iv 518

variations of . . . xiii 109

varieties of . . . ii 91

various forms of . . vii 483

with sloughing . . x 579

(see Fever, puerperal) . vii 483

inflammation, treatment of . vii 63

use of ice in . . . xiii 120

metastatic carditis . . xiii 119

inflammations . . xiii 116

ophthalmia . . . xiii 117

phrenitis . . . xiii 117

miasm and mephitis . . xiii 102

nervous irritability . . . iv 398

peritonitis, anatomy of . . xiii 107

bran poultice in . . vii 496

effects of . . . xiii 107

linseed-meal poultice in . vii 496

treatment of . . . xiii 108

use of ice in . . . xiii 108

phlebitis, cause of . . xiii 115

treatment of . . . xiii 120

practice, responsibilities of . vii 495

purulent deposits . . . xiii 116

putrilage, absorption of . xiii 116, 123

state, danger of senna and salts in vii 493

liable to rheumatism . xviii 522

treatment of . . . iv 399

sweating, Osiander on . . iv 399

women, typhoid fever in . xix 236

nephritis in . . . viii 156

Pullino, Dr., on cantharides . ii 549

Püllna, mineral water of . . xiv 345

Pulmo-cardiac function of Dr. Wardrop v 150

Pulmonary affections, creosote in . iii 509

tar-water in . . iii 509

use of cold in . . ii 531

apoplexy, Dr. Graves on . xvi 248

remarks on . . . viii 333

seat of . . . xv 88

source of . . . xi 408

treatment of . . . xvi 249

arteries, obstruction of . . xxiii 413

artery, four valves in a . . xv 154

laceration of . . xiv 547

obstructions in . . xx 93

second sound in . . xiv 178

transposition of . . xv 147

ulceration of . . xviii 367

atrophy, in the old . . vii 519

calculus . . . . viii 279

cavities, distinction of . . xvi 427

cavity, piece of lung in . . xvi 427

circulation, obstruction of . xxi 266

congestion, effects of . . vii 128

consumption, Dr. Clark on . i 70, 84

Mr. Gilbert on . . xiv 536

researches in . . xiv 225

crumpling sound in . . ix 313

disease, from swallowing a pin xix 30

worms, case of . xix 30

Pulmonary diseases—on climate in . xiii 423

comparative rarity of, in the navy xv 11

in West Indies . . viii 219

statistics of . . . xiii 423

emphysema . . . . vi 28

causes of . . . xi 406

peculiarity of . . xi 406

queries on . . . xviii 162

exhalation, crises by . . i 246

Tiedemann on . . i 241

hemorrhage, use of cold in . ii 532

hydatids, case of . . . xx 253

morbid microscopy . . xxiv 123

parenchyma, notices of . . xxiii 430

phthisis, in children . . xvii 310

phlebitis, case of, by Dr. Lee . ii 163

substance, degeneration of . xv 431

tests of infanticide . . vi 66

tubercle, Dr. Addison on . xxiii 423

tubercles, Dr. Kingston on . iv 151

tuberculous products . . xxiv 127

ulcer, cured by creosote . ii 168

vesicles, dilatation of . . vi 29

Pulmonic affections in United States xiii 426

pulse, described . . . iv 241

Pulsation, abdominal, Mr. Crisp on xxiv 413

epigastric, causes of . vii 511, 512

Dr. Hohnbaum on . . vii 511

diagnosis in . . . vii 511

Mr. Fausset on . . vii 513

treatment of . . . vii 512

in insanity, on discordant . xxii 13

jugular . . . . . . xix 105

of fœtal heart . . . i 89

of the heart, cause of the sound of i 437

Pulsations of the heart, Dr. Knox on iv 241

Pulsatory vesicles in reptiles . . iv 339

Pulse, the, and respiration, in old women i 555

and temperature, relation of . ii 246

at different ages . . . iv 241, 242

Dr. Guy on . x 288

at different times of day . iv 242

deceptive, in diseases of heart iii 434

diurnal variations of . . ix 376

effect of posture on . . vii 458

sleep on . . . iv 242

effects of exercise on . . iv 242

position on . . vi 134

experiments on . . . vii 461

fœtal, first detection of . . viii 368

in acute rheumatism . . ix 485

apoplexy, an unsafe guide . xxii 423

different postures . . iv 242, 243

diseases of the heart viii 456, 465, ix 485

gangrenous inflammation . ii 29

insanity, observations on . xxii 12

old age, cause of its force . vi 48

phthisis, Dr. Guy on . . ix 337

pregnant women . . vii 460

relation to inspiration . iv 242

its frequency in infants . . vii 76

its great importance in Turkey iv 387

jarring, in old age . . . viii 504

Pulse, more frequent in old age . i 556

M. Piorry on . . . vi 134

Mr. King's lever for . . v 333

no data for an average . . iv 242

of children, variableness of . viii 367

fœtus, unaffected by mothers viii 367

infants at the breast . . xiii 547

M. Valleix on . . xix 390

the old, M. Hourmann on . i 555

rapidity of, in fever . . vi 134

remarks on the fœtal . . viii 367

the pulmonic, described . iv 241

slow, in fainting fits, case of . xiv 55

state of, in sanguineous apoplexy xxii 388

vaginal, a sign of pregnancy . iii 248

variations of . . . . iv 241

in labour . . . vii 230

venous, Mr. King on a . . iv 365

Pulses of diseases of heart, table of viii 466

infants, tables of the . v 268

Pulticuli, or common graves . xv 522

Pulv. ipecac. comp., frightful doses of iv 118

Pump-suction in strangulated hernia i 586

Puncture, exploratory, in liver abscess xii 89

of bladder, researches on . xiii 241

Punctures in dissection, treatment of xi 310

Puncturing, in strangulated hernia, danger of . . . ii 201

the bladder, remarks on . v 467

Punishment, for crime, injustice of xvi 95

of death, remarks on . . xxiv 237

military, reasons for delay of . xxiii 165

surgeons' duty in . . . xxiii 164

on the ends of . . . xxiv 230

on vindictive . . . . xxiv 241

Punishments, effect of civilization on xviii 243

of soldiers, remarks on . . xix 388

remarks on . . . . . iv 191

Pupil, anomalies in the, explained . v 513

artificial, formation of . . xxiv 369

Mr. Jeaffreson on . . xviii 412

Mr. Tyrrell on . . xi 87-9

operation for . . . xi 194

Cheselden's operation for artificial x 39

dilatation of, by belladonna in the nose ii 263

green colour of, remarks on . ii 268

in typhus, Dr. Graves on . vi 554

on action of . . . . v 512

reflection of light in . . xvi 111

situation and action of . . x 10

Purgative, curious Russian . . xiii 69

Purgative action of hydropathy . xxii 452

draught, a useful occasional . x 47

medicines, abuse of . . viii 496

school, responsibility of . . v 390

-pill trade . . . . . vii 478

Purgatives, abuse of . . . x 245

after labour, opinions on . iv 400

cause of popularity of . . vii 477

Chanticleer's horror of . . vii 480

common abuse of, by women . ii 389

employment of . . . x 40

endermic use of . . v 352

Purgatives, endosmose in relation to xxiii 390

French horror of . . . x 65

griping, improper after labour ii 94

habitual use of, injurious . vii 477

in bilious disorders . vii 477, 480

in fever, M. Chomel's neglect of ii 63

remarks on . . . ii 63

mode of action of . . . xxi 162

Mr. Mayo on . . . . v 391-92

observations on . . . xvi 192

uniformity in . . . xxii 453

with antispasmodics . . xvi 200

narcotics . . . xvi 200

tonics, use of . . . v 391

Puriform matter in abdomen . . iv 48

PURKINJE, and VALENTIN, on ciliary motions in animals . . i 509

on ciliary motions . . iii 501

artificial digestion . . vi 527

the ovum . . . ix 5

vesicle of, in ovipari . . i 611

Purpura febrilis, fatal case of . xx 270

hemorrhagica, the blood in . xvii 150

Purpura, saline treatment of . . iii 263

some remarks on . . vi 428

Purpurine, and its chemical pathology xx 62, 64

Purring tremor, Dr. Hope on . viii 462

Purulent deposition in joints . . vii 149

deposits, Dr. Carswell on . i 149

from amputations . . xi 462

in distant parts . . xv 131

Mr. Mayo on . . . iii 69

rare in the kidneys . . ii 11

secondary . . . iii 18

remarks on . . ii 11

diathesis, M. Bonnet on . xxii 63

expectoration, on great . . xi 408

infection of blood, Dr. Taylor on xxiv 562

infection, theory of. . . . xx 218

treatment of . . . vii 498

metastasis, Dr. Froriep on . i 569

ophthalmia of infants . . xviii 410

urine . . . . . vi 142, 135-6

in hepatic abscess . . xxiii 149

Pus, absorption of, and discharge of, with urine . . . . xvii 440

action of, on the blood . . xvii 148

analysis of . . . . . xii 136

and mucus, good criterion of . xvi 147

Mr. Addison on . . xviii 95-6

and its corpuscles, M. Buhlmann on . . . . . xvii 519

constituents of . . . . xxii 22

and properties of . . xvii 444

deposits of, after labour . . viii 56

causes of . . . . xv 131

distinctive characters of . . iii 530

Dr. Carswell on . . . i 147

Dr. Donné on the properties of iii 531

effect of ammonia on . . iii 531

effects of absorption of . . xviii 112

evacuations of, in hepatitis . vi 251

formation of . . . . xviii 111

Pus, from liver, excreted by other organs viii 139

healthy, unirritating . . xv 46

how distinguished from mucus iii 531

in coagula of the heart . . i 148-9

in contact with bone, effect of v 251

injected into veins of animals . iii 506

injection of into veins, effects of xv 131

in milk, discovered with difficulty vi 185

in the blood . . . . vi 136

mode of detecting iv 526

in the urine, cases of . . vi 135-36

microscopical examination of . vi 546

Mr. Mayo on the formation of iii 62

globules in . v 112

Mr. Travers on the nature of . xviii 75

nature and operation of . . xiii 336

normal, diagnosis of . . xxii 332

observations on, by Dr. Davy . xii 135

on its recognition in the blood, &c. iii 530

on vessels in surfaces secreting xi 451

pathology of . . . . xxii 330

peculiar action of ammonia on viii 148

test of, its burning with a flame xvi 147

the consequence of low vitality xviii 75

unrestricted effusion of . . xviii 112

Pus-cells, fibrous tissue and tubercle from xx 122

Pus-corpuscle, structure of, Vogel on xxii 330

infusoria in . . . xxii 332

proper . . . . xxii 21

genesis of . . . xviii 276

Mr. Addison on . . xvii 93

Mr. T. W. Jones on . xviii 275

Pus-globules, chemical tests of . vi 185

contain a nucleus . . ix 509

dissolved by bile . xvi 147-48

Pustulæ, syphilitic history of . . xxiii 355

Pustula maligna, cause of . . vii 550

description of . . vii 550

treatment of . . vii 550

Pustular eruptions, artificial . . xv 390

infantile . . . vii 93

Pustule, malignant, diagnosis of . xiii 42, 44

in animals . . xxiv 86

in man . . . xiii 42

not infectious . . xiii 45

of eyelids . . x 26

Pustules, artificial, inoculation from xv 391

classification of . . . xv 388

from animal matters . . xv 891

PUTNAM, Dr., his translation of Louis iii 456

Putrefaction, aided by hydrophosphoric acid . . . ii 396

brown . . . . . . ii 398

changes in tissues by . . viii 123

corrosions in . . . . ii 399

delayed by a stream . . ii 397

desiccation in . . . ii 399

different media favouring . ii 400

evolution of gas in . . . ii 398, 412

favoured by aqueous vapour . ii 397

by nitrogen . . . ii 396

green . . . . . . ii 397

hastened by electricity . . ii 397

Putrefaction, incrustations in . . ii 399

in relation to legal medicine . ii 396

in spring . . . . . ii 402

in summer and winter . . ii 400-1

in water, hastened by heat . ii 400

quicker in summer . . ii 400

what modifies . . . ii 399

observations on . . . xvi 196

occurs in vacuo . . . ii 396

of animal substances . . xix 252

of dead bodies, time of . . xv 519

oxygen gas conducive to . ii 396

prevention of . . . . xvi 196

production of . . . xi 135

Professor Devergie on . . ii 396

retarded by azote . . . ii 396

by carbonic acid . . ii 397

chloride of calcium . ii 397

chlorine . . . ii 397

deutoxide of nitrogen . ii 397

hydrogen . . . ii 397

nitrous acid . . ii 397

sulphurous acid . . ii 397

slow in privies . . . ii 396, 400

three kinds of . . . xiv 449

under water, Devergie on . ii 397

table of progress of . ii 400

Putrescence of the uterus, cases of . iii 246

Putrescent exhalations, innocency of xi 13-19

Putrid children, danger of turning . ii 80

decomposition, products of . xxiv 249

diseases, observations on . xvi 196

effluvia at Montfaucon xi 14 (App.) 39

Dr. S. Smith on . . xi 9-13

effects of . . . xi 9-19

fever from v 271, (App. to vol.) xi 37

substances absorbing (App. to) xi 71

with marsh miasmata . xi 21

emanations, injurious to health xv 515

insalubrity of . . . xvii 404

noxious or not . . xiv 449

matter injected into veins . iv 164

inoculation with (App. to vol.) xi 37

meat, poisoning from . . xv 238

water, effect of injecting . viii 316

Putrilage, progress of . . . ii 398

puerperal, absorption of . xiii 116, 123

Pyelitis, acute, characters of . . xv 471

calculous . . . . xv 472

cancerous and tuberculous xv 472, 480

chronic, characters of . . xv 471

diagnosis of . . . . viii 125

incorrectness of, Dr. Prout on xi 359

M. Rayer on . . . . xv 470

nephrotomy in . . . xv 475

pathology and diagnosis of . xv 471-5

species of . . . . viii 150

treatment of . . . . xv 475

ulcerations in . . . xv 472

Pyelo-nephritis, meaning of the term viii 150

M. Rayer on . . . xv 476

Pyin, Prof. Vogel on . . . xxii 332

Pylorus, in poisoning by mineral acids iii 540

Pylorus, scirrhous, acid secretions in xiv 576

Pym, Sir W., on Alexandria and Cairo xxiv 244

Pyoid corpuscle, Dr. Lebert on . xxii 22

Pyrenees, springs of the . . xiv 353

Pyrexia, Dr. Clutterbuck on . . v 185

in maniacal cases, Jacobi on . xxii 13

Pyrmont, the mineral water at . xiv 355

Pyromania, jurisprudence of . . x 170

in young females . . . x 170

Pyrosis, and gastrodynia, diet in . x 43

treatment of . . . x 43

azotic food in . . . xii 506

case of . . . . . xx 239

diagnosis of . . . . xii 505

Dr. West on . . . . xii 503

from pressure . . . xiii 558

indications in treatment of . xii 506

nature and treatment of . ix 273

or water-brash, theory of . xii 503

symptoms of . . . . xii 505

treatment of . . . . xxiv 142

use of nux vomica in . . iv 244

usual causes of . . . xii 505

Pyrotic fluid, on the source of the . xii 504

Pyroxylic spirit as an antiseptic . ix 397

Pythagoras, account of . . xxiii 91

an inductive philosopher . xviii 170

his philosophy of music . . xviii 170

mysticism of . . . . xviii 171

philosophy of . . . v 82

Pythagorean imitators as to food . xxiii 496

secret societies . . . xxiii 91

Quackery, Dr. Crowther on . . vii 49
Dr. M. Hall on . . . xxiii 196
in America, rules on . . iii 278
England, Dr. Marx on . xv 23
medical men abettors of xxi 514, 533
report on . . . . viii 610
Quack medicines, on the success of xxii 562
Quacks, and quackery, remarks on xvii 193
aristocratic patronage of . xxi 533, 535
reflections on the success of . xiv 422, 426
Quakers, frequency of insanity among xv 55
mean age at death of . . xviii 505
Quain, Dr., his Elements of Anatomy v 216, xxi 499, xxiv 386
Quain, Mr., his great work on the arteries xi 210, xix 486, xxii 556
comparison of . . xix 505
Qualification in medicine, on one . xiv 411
Quality, alterations of, in the body xxiii 51
Quarantine, abolition of . . xvi 305
a medical commission on . xvi 305
and plague, works on . . xvi 289-90
Dr. Bowring on . . . vi 582
improved, benefits from . . xvi 307
improvement of . . xvi 305, 307
laws, consideration of . . xix 46, 49
correspondence on . . xxiv 242
Mr. Holroyd on . . xvi 290

Quarantine, on periods of . xxiv 257, 260

regulation, foundation of . xvi 301

regulations on, English . . xxiv 263

French . . xxiv 262

regulations, objections to . viii 235

works on . . . . . xix 41

Quarantines, and plague, Dr. Bowring on viii 233

inefficiency of, in cholera . xxiii 330

Quarriers, phthisis of . . . xx 252

Quartering of troops in West Indies xxi 174

Quassia, poisonous to flies . . iv 130

Queen Adelaide Hanwell fund . xi 271

Queen, medical staff of the . . iv 568

Queen's medical staff, remarks on the v 548

QUETELET, M., his Essay on Man . iii 40

his tables of mortality . . iii 53

on periodic phenomena . xviii 165, 177

Quickening, Dr. Davis on . . iii 406

Dr. Montgomery on . . iv 453

Quicksilver, cases for exhibition of viii 422

in introsusception . . . viii 422

QUIN, Mr., his Pharmacopœia Homœopathica . . . xxi 225

Quina, mode of disguising the taste of iv 246

salts of, endermic use of . . v 350

use of the salts of, remarks on xvi 200

Quinary system of animated nature xix 181

Quinina, hydrocyanoferrate of, in fever x 584

Quinine and iron mixture for ague xvi 562

Quinine, deafness and dumbness from x 586

diminution of spleen by . . vi 141

disulphate of, poisonous effects of xviii 164

endermic use of, in ague . ii 178

ulceration by . ii 178

in acute rheumatism . . xx 268

agues, modes of prescribing iii 105

asthma, use of . . . xv 239

cerebral diseases . . vi 145

diseases of children . . xiv 560

of the eye . . x 8

fever in India . . . xxiii 118

fevers, remarks on . . xiii 560

intermittent epistaxis . . iii 511

intermittents . . . viii 61, 64

M. Melier on . . xviii 162

nervous diseases . . . vi 145

yellow fever . . . x 278

its effect in ague . . . xii 94

mode of disguising . . xiv 560

new mode of exhibiting . . xv 236

salicine a substitute for . . i 570

sulphate of, in typhoid fever . xv 235

on prescribing . . xii 569

Quinsy, on alum gargles in . . vii 551

scarifications in . . . vii 550

two kinds of . . . . vii 550

Rabies canina in animals . . xxiv 90
Rabies, in animals, Dr. Wagner on v 262
in the dog and ox . . . v 262, 263
horse, pig, and sheep . v 265
Rabies, transmission of . . xi 229
Races, extinction of . . . xx 154
of mankind, common origin of xxiv 49
observations on xviii 387, xxiv 218
Rachitis, case of congenital . . xx 549
Dr. Elsässar on . . . xvii 376
erratic character of . . xvii 377
infants not free from . . xvii 376
limited to the skull . . xvii 377
thoracic, queries on . . v 139
RACIBORSKI, M., his errors on phthisis i 488
his Manual of Auscultation . i 479
on puberty . . . . xix 84
remarks on his style, &c. . i 489
Radcliffe fellowship of Oxford, on the xxii 119
Radesyge, case of . . . iv 229
Dr. Hjort on . . . . xiii 461
RADFORD, Mr., on inversion of the uterus . . . . . v 288
Radiata, nervous system of the . v 494
Radiated tribes, formation of the . xv 267
Radical diseases of Dr. Lanza . xxiii 60
Radicals, compound, in chemistry . xi 132
Radius, dislocation of, forwards . xiv 42
fracture of the carpal end . ii 256
of lower end of . xiv 230, xv 242
of, remark on . . vi 309
fractures of, Lisfranc on . xiv 6
Rain-sound in the ear . . . viii 99
RAINY, Dr. H., on urea in the blood vii 586
RAINEY, Mr. G., on the arachnoid membrane . . . . xxiv 324
on structure of lungs xxi 552, xxiii 415
Rambles in Europe, Dr. Gibson's . xii 232
Ramollissement, Dr. Carswell on . i 137
of the brain . . xi 464, xiii 535
remarks on . . . iii 312
of the heart . . . . ii 330
RAMSBOTHAM, Dr., his Midwifery xii 461, xv 527
his obstetric plates . . ix 540
on obstetric medicine . . xix 235
RANKIN, Dr., on the Pali plague . ix 234
RANKING, Dr., his translation of Lugol xix 239
Ranula, fluid of, composition of . xv 236
instrument for . . . vi 164
nature of . . . . xx 134
Sir C. Bell on . . . vi 164
Ranunculaceæ, Dr. Turnbull on the ii 499
Ranunculus, action of varieties of xiii 232
Rape, case of, at Derby Assizes . xix 39
conception after . . . vii 374
Raphania, from eating spurred rye . xvii 421
Rapport magnétique, remarks on . xix 479
Rarefaction of blood, its effect on sound v 168
RASCHKOW, M., on the teeth . viii 158
Rash, the typhoid . . . viii 432, 436
RASORI and GIACOMINI, doctrines of xxi 159
RASORI, biographical sketch of . vi 289
comparison of with Hunter . vii 426
dangerous practice of . . xv 462
his claim to first using large doses of emetic tartar . i 416
contra-stimulant doctrine . vii 425

Rasori, his practice, as stated by himself i 418
his theory of inflammation vii 417, 424, viii 169, 202
obituary of . . . . vi 289
Rasorian doctrine declines in Italy . i 497
Raspail, a saying of . . . xix 180
his spiro-vesicular theory . iv 10, 11
system of botany . . iv 2, 3
vermicular theory . . xxii 546
on impregnation of plants . iv 29
the virtues of camphor . vii 549
shrewd precaution of . . x 75
strictures on his work . . iv 4
Ratier, M., on syphilis . . xxii 226
Ratio medendi, of Prof. Lanza . xxiii 49, 52
Rational pathology, observations on xxiv 295
soul, Stahl's theory of the . xxiii 585
Rations to troops in India, improved xxi 375, 376
Rats and moles eaten in China . xvii 16
Rattlesnake, fatal case of bite of . vii 578
Rau, Dr., on the mortality of infants vii 591
Ray, Dr., his jurisprudence of insanity x 129, 153, xix 33
Ray, electric, a cure for gout . . xi 233
Ray-liver oil, use of in scrofula . viii 554
Rays, non-luminous, relation of to eye xxii 287
on the swimming of . . xxii 537
Rayer, M., his Atlas of Illustrations ii 157
on diseases of the kidneys viii 121-57, x 301, 328, xv 470
skin . . ii 147
on glanders in man . vi 113, xiii 28
plates to his work on kidneys viii 157
remarks on his work . . ii 156
Rayer's ointment for tinea capitis i 578
Reaction, after loss of blood, nature of iii 326
and restitution . . . xxiv 299
in disease, Dr. Canstatt on . xiii 336
typhus fever . . . viii 445
many modes of, in living bodies i 391
Reading, aloud, utility of . . xvii 421
educating idiots in . . . xxiv 10
keeping up habits of . . xviii 234
Reason, the essence of all science xxiv 221
Reasoners, on a certain description of xxiii 163
Reasoning, and experiment, remarks on viii 352
aphorism of Coleridge on '. xxiv 220
with the insane, remarks on . xxi 463
mania, description of . . xx 28
Rébouteurs in Switzerland, account of vi 312
Récamier on nux vomica in diarrhœa i 257
Receptacles and bracts as food . xvii 20
Receptaculum chyli, remarks on the iv 344
Reclamation of Dr. Hamilton . iv 181
Recoara lithotriptic, fountain of . ix 246
Recovery from disease, Henle on . xxiv 318
Recreation of the insane . . xxi 463
Recruits from large towns, fewer . xv 307
manufacturing districts xxi 356
in India, mortality of, as to age xxiii 112
military, Dr. Jackson on . xxi 171
Recto-vaginal fistulæ, cases of . ii 201-2
operations for . xxi 322
Recto-vaginal malformation, case of xviii 453
septum, tumours in . . xviii 18
Rectum, and brain, different heat of xii 140
application of leeches within . xxiv 512
cancer of, extirpating . . xi 198
M. Vidal on . . xviii 452
carcinomatous stricture of v 485, xviii 452
chronic inflammation of . . v 485
ulcer of, like cancer . v 485
congenital septa in . . iv 468
danger of forcibly dilating . iv 471
Dr. Bushe on diseases of . iv 467
examination of females by the ii 514
expanding bougie for the . iv 471
foreign bodies in the, cases of iii 236
obstructing . . iv 468
imperforate, Dr. Bushe on . iv 468
new canal for . . . iv 468
Mr. Mayo on diseases of . iii 75
on stricture of . iii 76
Mr. Syme on diseases of . v 480
obstructed, case of . . . iv 469
on examination per . . iii 412
operations on . . . xxi 328
perforated by bougies, case of x 45
polypi of, in children . . xvii 561
purulent exudation from, case of xxiv 121
relaxation, &c. of, case of . iv 470
removal of part of . . . xii 256
scirrhus of, cured by excision . v 486
operation for . xii 256
seat of permanent stricture of iii 76
simple ulcer of, treatment of iii 75
spasmodic stricture of . . iii 76
spreading ulcer of . . . iii 75
stricture of, on bougies in . v 484
treatment of . . . xxi 329
tenesmus in obstructed . . x 48
terminating in urethra, case of xviii 456
Rectus femoris, rupture of, case of xix 247
Recurrent and rhythmical disease xxiv 317
nerves, asphyxia from section of iii 29
effect of section of . . iii 29
Red men of America . . . xxiv 473
people of Africa . . . xxiv 447
snow, or protococcus nivalis vii 176, 182
Redford, Mr., on the body and soul xxiv 287
Redness, from imbibition . . viii 195
inflammatory, forms of . . viii 193
of arteries after death . . ix 225
Reed, Dr., on fever, cholera, &c. xxiii 241
Rees, Dr., on conditions of the urine xii 346
on diseases of children xiii 219, xviii 236
earthy matter in bones . vii 154
mollities ossium . . ix 389
the blood . . . . xviii 336
and urine . . xxii 223
the liquor amnii . . vi 581
Rees, Dr. G. O., on the blood . xxi 544
Refinement, American precocity of v 410
Reflected irritation, definition of . ii 3
examples of . . ii 5
membranes, erysipelas of . ii 24

Reflection, aphorism of Coleridge on xxiv 220
Reflections and Notes, Dr. Holland's viii 482
Reflector epiglottidis muscle . . xiv 377
Reflex action—centric, Unzer on . xxiv 206
Dr. Laycock on . . xix 299
Dr. Stilling on . . xvii 392
laws of, Unzer on . . xxiv 201
M. Valentin on . . xi 300
of the nerves, Unzer on . xxiv 195
on distinct nerves for . xvii 181
practical use of . . xxiii 197
Prochaska on . . . xxiii 212
report on . . . xvii 264
without sensation . . xvi 160
actions, of lower animals . xxiv 204
requisites of . . . xi 453
vis nervosa stops . . xi 453
acts, three kinds of . . xxiv 208
doctrine, Dr. Arnold on the . xvi 158
doctrines propounded by Unzer xxiv 210
function, the, Dr. Arnold on . xvi 180
Dr. M. Hall on . . xvii 171
effect of volition on . xi 453
of the brain . . . xix 298
spinal cord . . iii 33, 577
Prochaska on . . . v 623
use of, in midwifery . xvii 179
motions, all involuntary . . . vi 216-17
Dr. Volkmann on . . vi 211
experiments on . . vi 212
extent of . . . vi 213
in decapitated frogs . vi 211
in relation to mind . . vi 217
modification of . . vi 214
of the sympathetic . . vi 214
prevented by the will . vi 216
sensation in . . . x 257
the different seats of . vi 213
nervous arcs, interruption of . xi 454
phenomena, analysis of, by Unzer xxiv 205
sensations and motions . . xxiv 311
theories, before Dr. Hall. . xvi 175
theory, Dr. Dowler on . . xxiv 280
sketch of . . . xvi 159
Reflection, of motions, Prof. Müller on v 536
signification of the term . . xvi 176
Reformation of medicine, suggestions for xxi 262
Reform, medical . . . ix 281-7, x 297
documents on . viii 294, 299
Dr. von Walther on xiv 402
essay on . . xix 193
in Germany . . xiv 403
in Spain . . i 608
memorial on . . xiii 580
petition for . . viii 612
remarks on . . viii 299
Sir J. Clark on xiv 296, xv 529
of London College of Surgeons ix 287-90
Refrigerants, Dr. Murray's theory on xvi 195
observations on . . ii 388, xvi 195
Refrigeration in diseases, remarks on xxii 448
Refuse of towns, money value of . xv 334
application of . xviii 495
Regimen, and diet, on popular works on xviii 115
and longevity, Dr. Bell on . xviii 115
benefit of attention to . . xiv 425
dietetic, in disease . . . xvii 51
homœopathic, observations on xxi 232
Regions, Dr. Lebaudy's anatomy of ii 196
Registers, mental and tabular . v 161
Registrar-General, his plan . . ix 348
his report for 1837-8 ix 344
for 1838-9 . xi 424
for 1838-40 . xv 285
for 1841 . xv 74
reports . . xviii 197
annual report . xxi 1
Registration Act, the new . . ii 611
account of English . . . ix 349
of births and deaths viii 532, xviii 205
of diseases, general . . iv 280
of practitioners . . . xix 203
Registry of prostitutes in Paris . iv 71
Regnoli on pulmonary vapour . i 241
on watery vapour from the lungs i 241
Regurgitation of stomach, morbid xx 238
Reich, Prof., on the fever of growth ii 219
Reid, Dr., on perception and sensation iv 446
Reid, Dr. D. B., construction of his
class-room . . . . xix 20
on ventilation . . . xvii 532
warming and ventilation . xix 1
Reid, Dr. James, his Manual of Midwifery i 512
Reid, Dr. John, on aneurisms of aorta ix 571
on asphyxia . . . . xii 278
muscular contractility . xii 277
structure of the placenta . xi 540
the eighth pair of nerves v 308, vi 574,
viii 264
the functions of the nerves ii 600
the ganglionic nerves . viii 594
the respiration . . . vi 580
typhus fever . . xii 293, 313
Reid, Dr. W., on practice of medicine ix 461, 494
Reil, his theory of life . . . v 78
Professor, on medical reform xiv 403
on the cerebellum tiresome &c. iv 487
Reinsch, M., his new test for arsenic xvi 275
his process for arsenic, remarks on xx 330
Rejuvenescence, Dr. Mehliss on . vi 75, 80
fatal results of . . . vi 80
on human . . . . xvi 208
table of . . . . . vi 80
Relapse-periods in agues, remarks on xxi 529
Religion, and electricity, Dr. Dickson on xv 129
and mortality of medical men xxi 438, 442
physical temperament xv 414, 426, 429
charity in, remarks on . . xiii 419
cruelties from false views of . iv 56
its influence on health . . iv 55
Religious beliefs, affinity traced by xxiv 459
conversion by whipping . . iv 57
excitement in America . . iv 58
exercises of the insane . . xxi 464
insanity, Mr. Newnham on xv 422, 429
instruction to the insane . xii 54

Religious observances by the insane xxiv 164
services to the insane . . xix 339
REMACLE, M., on foundling hospitals xiii 289, 293
REMAK, Dr., his investigations . xxiii 503
on blood-globules . . . xiii 229
menstrual blood . . ix 545
new nervous ganglia . . xv 232
structure of the nerves vi 394, vii 500
Remedies, absorption of, Dr. Paine on xv 147
action of, view of . . . xiv 330
agreement or tolerance of . xxiii 44
American, odd . . . xix 29
and poisons, on the action of xiv 520
changes of, in living body . viii 354
circumspection in the use of . xxi 450
effect of . . . . . viii 355
exaggerated praise of . . xvi 188
key to the operation of many . xiv 516
local effects of . . . xi 454
maxims on the use of . . xxiii 50
mesmeric instinct of . . xix 448
mixture of similar . . . xvi 198
new, Dr. Dunglison on . . ix 541
remarks on the neglect of . iv 249
use of new and old . . xxii 171
Remedial means, on forbidden . xxi 451
Remembrancer, Medical . . xix 246
Remittent and yellow fever, diagnosis of xvii 365
different xvii 368
fever, American, Dr. Currie on xvii 365
Dr. Bartlett on . . xvii 357
in children, report on . xvii 563
morbid anatomy of xvii 363, 367
of children . . . xvii 563
of India, account of . iii 349
Remora, median ganglion of . . xxii 498
Renal and other tumours, diagnosis of xv 475
and stomach diseases, Dr. Prout on xvi 477
apoplexy, M. Rayer on . . xv 478
capsules, report on . . . xxi 567
disease, and diabetes, compared ii 227
character of œdema in . x 323
Dr. Bright on . . viii 121, x 301
Dr. Prout on varieties of . xi 347
influence of age on . . xi 346
in the fœtus . . . xx 548
of Dr. Bright, disquisition on ii 225
pericarditis from . . xxiv 557
with dropsy, cases of . ii 226, 228
with irritable bladder . xi 360
treatment of xi 362
diseases, caution on . . xi 351
classification of . . viii 124
in relation to the brain . xxii 427
percussion in . . . viii 125
prognosis in . . . xi 350
treatment of . . . xi 350
dropsy, cause of . . . xii 119
M. Rayer on . . . xv 484
fistula, M. Rayer on . . xv 477
granulations, structure of . xxii 26
hemorrhage, idiopathic . . xv 481
M. Rayer on . . . xv 478
Renal hemorrhage, singular case of xv 479
inflammation, arthritic . . x 303
organs, mechanical lesions of . viii 149
Renewal, healthy and morbid . . xvi 216
process of, cultivation of . . xvi 216
RENOUARD, M., his History of Medicine xxiii 86
Reorganization, process of . . xvi 209
Reparation by natural growth . . vii 426
in the lowest animals . . vii 426
Reparatory process, not inflammatory vii 426
Report on anatomy and physiology . xxii 261
on arsenic . . . . . xvi 275
midwifery, by Dr. West . xxiii 277
the ovum . . . . xvi 513
Persian ague . . xvi 558
progress of medicine, &c. xx 209
Reports, plan for improved medical . vi 381
select clinical . . . . xxiii 419
Repose, and equilibrium in nature . xxi 117
necessity for periods of . . iii 163
Repository, general medical (German) i 220
Representation of the profession . xix 216, 550
Reproduction and nutrition . . vii 179
of parts . . xi 304
in vegetables . . . . vii 182
observations on . . . ix 2
of lost parts . . . . xx 307
Mr. Newport on . xx 504
Reproductive organs, in vegetables . vii 183
relations of . . xii 61
power in man and animals . vii 387
process in plants . . . iv 561
Reptiles, cerebellum in . . . xxii 538
crocodiles, and snakes, as food xvii 16
heat in incubation of . . xii 529
teeth of . . . . . . xx 398
attachment of . . xx 399
temperature of . . . xii 143
Resection of bones, tetanus after . xx 51
of large joints, M. Bonnet on . xxii 83
Resemblance of children to parents . vii 375
Resolution of inflammation . . xviii 279
Research, on reforming the modes of xxiii 161
Respiration, abdominal, in males . xvii 254
action of diaphragm in . . xvii 256
affected by cutaneous nerves . v 512
darkness . . v 33
after birth, cause of first . . xiv 223
death, remarks on . . v 104
air-changes in . . . xxi 553
air of, quantities of . . xvii 152
ancient physiology of . . xv 448
and animal heat, report on . xiv 297
circulation, relations of . vii 263
temperature, relation of, to pulse ii 246
artificial, in poisoning with opium iv 142
before entire birth . . . xvi 70
Borelli's view of . . . v 135
Brachet's doctrine of . . xiii 223
by the blood-vessels . . xvi 215
capacity of . . . . xix 264
causes of frequent . . . vii 264
changes of air in . . . xix 265

Respiration, changes of blood in . viii 480

cutaneous, importance of . . xxii 190

different opinions on . . v 511

diseases of the organs of . . xxiii 303

Dr. Schultz's theory of . . xvi 215

exaggerated . . . . ix 312

experiment by Dr. Hall on . xvii 180

first, truly explained . . i 383

in early infancy, state of . . xx 358

inferior costal, in animals . xvii 254

in regard to infanticide . . ii 409

in relation to appetite . . xix 10

to heat . . . xi 394

to the pulse . . . iv 242

in sleep, on promoting . . vi 584

in winter-sleep . . . xxiv 408

its effects on the blood . . xvii 484

Liebig's theory of . . . xiv 521

medulla oblongata in . . xiii 224

mode of investigating . . v 144

motions of . . . . xiii 223

movements in . . . . v 511

M. Beau on the sounds of . vi 584

M. Valentin on . . . xi 296, 303

Mr. Hutchinson on . . . xxiv 167

Mr. Jeffreys on . . . xvii 152

Mr. Paget's report on . . xxi 552

nerves affecting . . . viii 265

normal and abnormal . . ix 304

of child during birth . . iv 101

of insects, Mr. Newport on . iii 291

of plants . . . iv 22, 24, v 382

of the old, M. Hourmann on . i 553

organic and involuntary . . iv 421

present knowledge of . . xix 263

process of, reflections on . xxi 112

puerile . . . . . ix 311

cause of . . . . i 481

quantity of air used in . . vi 580

report on . . . . xvii 254

Sir C. Bell's opinions on . iv 422

sounds of . . . . vi 584

state of, in phthisis . . ix 329

superior costal in females . xvii 254

supplementary . . . ix 311, 312

table of morbid . . . ix 319

the great end of . . . xvi 226

theory of, Dr. Stevens on the . ii 216

volition in . . . . iv 421

relation to . . xi 456

use of controlling . . . v 151

uterine, remarks on . . ii 409

Respirator, good effects of the . x 591

Mr. Jeffrey's, principle of . iii 493

notice of the . . . ii 373

Respiratory anæsthesia . . xvii 419

functions, Mr. Hutchins on . xxiv 327

ganglia, case of sleep of . xix 306

motions, Dr. Hall on . . xiii 223

Dr. Reid on . . . xiii 225

movements, account of . . xix 263

force of . . . . xix 264

report on . . . xvii 254

Respiratory murmur, absence of . vi 136

murmurs, comparison of . ix 307

on the two . . . ix 300

reckoning of . . . ix 305

theories of . . . xix 106

muscles, action of . . . xvii 255

spasm of . . . xvii 420

nerves, Dr. Arnold on . . viii 480

in medulla oblongata . xviii 136

of animals . . . viii 510

Sir C. Bell on . . viii 480

organs, affections of . . x 583

diseases of, in animals . xxiv 84

in England . . xi 425

inflammatory products of xxiv 123

Mr. Wardrop on . . ii 218

vibratory motions in . i 510

sounds, properties of . . ix 302

spasms, reading aloud for . xvii 421

system, J. Hunter on . . xv 208

motions of . . . xi 296

vesicular sounds . . . ix 304

Responsibility, criminal, and insanity xxiv 226

in flogging in the army . . xxiii 165

law of . . . . . xvi 107, 273

legal, treatise on . . . xiv 529

of practitioners . . . i 306

Rest-harrow, a cure for rheumatism x 558

use of, in rheumatism . x 558

Restiform body, gray nucleus of . xxiv 575

Restrained madman, rupture of spine in a xiii 542

Restraint, in insanity, Dr. Steward on xxii 61

evil effects of . . xiii 275

in lunatic asylums . . . xi 105

of lunatics, pleas for . . xix 607

total disuse of . . xv 218

of the insane, considered . ix 154

or non-restraint in insanity xix 297, 334, 598

Resuscitation, after interment . v 582

of a newborn infant . . v 512

Rete mucosum . . . . ii 446, 452

Mr. Glover on the . . vi 574

Retention of urine, hysterical . iv 136

Retina, the, action of . . . x 13

anatomy of the . . . iv 499

component elements of . . vi 422

four layers of . . . vii 281

inflammation of . . xx 282, 510

intimate structure of . vi 422, x 12

macula lutea in . . . v 298

Michaelis on . . . . v 298

minute anatomy of . . . v 296

of the cuttlefish, Mr. Jones on vi 421

ossification of, nature of . xiii 506

papillæ in . . . . vi 420

partial paralysis of . . xvi 254

peculiar structure of, view of . xviii 143

perception of colours by single x 20

relation of its coats . . v 297

structure of . . . xxii 286

Treviranus on . vi 419, 420, v 297

true sentient, extent of . . xxiv 367

tumour in front of, account of xxii 31

Retinæ, correspondence of the two . x 19
corresponding points of . . x 19
Retinitis, chronic, cases of . . vi 74
objection to the term . . v 43, 44
Retired man of business, his evils . i 340
Retreat, the, at York, for the insane, state of vii 2, 53
for the insane, at York . ix 144
history and statistics of . . xxii 363
Midland, notice of . . i 308
on the use of restraint in . xxii 364
statistics of . . . . . xiii 213
Retreats, convalescent, in India . i 345
Retrograde action of stimuli on nerves xi 455
Retro-pharyngeal abscess, case of . xx 558
report on . xvii 558
Retrovaccination, directions for . ix 85
discussion on . . ix 82
experiments on . . xx 563
Mr. Ceely on . . x 468
Retroversio uteri, case of . . vii 451
catheter in . . iv 410
treatment of . . iv 410
Retroversion of unimpregnated uterus xii 262
of uterus . . . . . x 286
cases of . . . v 577, viii 54
Dr. Ashwell on . . xix 357
Lisfranc on . . . xviii 24
on puncturing . . iv 411
puncture in . . . viii 55
reduction of . . . xviii 25
Retroverted uterus, instrument for . iii 406
mode of replacing . xix 422
Returns, on military medical . xvi 34
public, on nosology in . . xxiii 441
RETZIUS, Prof., his preparations of
the heart . . . . . iii 506
on catamenial blood . . ii 274
structure of the brain . xxii 502
the form of skulls . . xviii 372
Revaccination, at the Foundling Hospital vii 581
Dr. Gregory on . . . xii 97
failures of protection by . . vii 210
in the Danish army . v 209, ix 292
Hanoverian army . . xiii 551
India . . . . . xx 354
Prussian army ii 280, v 210, vii 567,
viii 261, x 276, xiii 550, xiv 237
Wirtemberg . . vii 186 203
marks at, and after . . vii 205
matter, value of . . . vii 208
modifications in . . . vii 207
Mr. Newnham, &c., on . . viii 546
protection by . . vii 206, 209
proper age for . . . vii 206
pustules of, Dr. Hoefle on . xxiii 308
varieties of . . vii 207
report on . . xvii 563, xx 562
results of . . . vii 205, 206
the French Academy on . vii 567
Review, British and Foreign Medical xvi 490
animus of the criticised towards the xxiv 593
arrangements of the . . xxiv 586
circulation and success of the xxiv 590, 591
due economy in relation to the xxiv 595
Review, British and Foreign Medical—
Editor's small literary share in xxiv 590
impediments to sale of the . xxiv 591
its excellencies against its sale xxiv 593
judgments and criticisms of the xxiv 590
leading principles of the . . xxiv 586
need for establishing the . xxiv 586
number of copies of, printed . xxiv 588
on a quarterly rival of the . xxiv 592
on homœopathy, cry against the xxiv 594
reasons for relinquishing the . xxiv 590
remuneration for articles in the xxiv 586
to contributors to the . xxiv 595
sale of the . . . xxiv 589, 591
success and patronage of the . xxiv 589
Review, Rio Medical . . . ii 234
Reviews, journals, and transactions xxi 373
medical, former deficiencies in xxiv 585
on gratuitous contributions to xxiv 585
Reviewer, a disagreeable task to a . vi 498
Reviewers, gutting school of . . xvii 119
Revivifying remedies, Dr. Lanza on xxiii 49
Revue Médicale Française . . i 225
Revulsion, and derivation, remarks on ii 173
Dr. Rognetta on . . . xxi 162
Wiseman on the term . . vii 57, 58
Revulsive bloodletting . . . i 496
treatment, remarks on . xv 407, 409
Reunion, and reorganization, modes of vii 429
of parts, Prof. Burns on . vii 434
REYBARD, M., his canula for paracentesis xiii 371
REYNOLDS, Dr., his Aretæus . . iv 490
RHAZES, on choice of a medical attendant xxiii 96
Rheims, typhus at, in 1833 and 1840 xiv 224
typhus fever at, symptoms of . xvii 359
Rheumatic affections, of the heart . xiii 452
sulphur in . . x 586
anomalous ailments . . xiv 413
deformity, myotomy in . . xiii 566
diathesis, Dr. Todd on . . xvi 468
M. Bonnet on . . xxii 63
diseases, Dr. Greiner on xiv 412, 421
statistics of . . . xiii 423
fever, bleeding in . . xiii 449, 452
calomel in . . . xiii 451
cinchona in . . . xiii 451
diaphoretics in . . xiii 451
opium in . . . xiii 451
or acute rheumatism . xiii 446
purgatives in . . . xiii 450
treatment of . . . xiii 449
gout . . . . . . vi 378
root of colchicum in . vi 556
knobby disease, in Turkey . iv 393
neuralgia, diagnosis of . . xiv 412
ophthalmia, treatment of . xv 403
pericarditis, treatment of . xx 189
swellings, use of iodine in . viii 523
Rheumatism, abdominal, chief sign of vi 357
obstinacy of . vi 357
treatment of . vi 357
aconite in . . . . . xx 467
active chronic, treatment of . iii 555
acute, anatomy of . . . xiii 447

Rheumatism, acute—bleeding in . vi 367

Bouillaud on . . . v 230

cases of . . . v 231

colchicum in . . . vi 369

danger of bleeding in . v 231

duration of . . . vi 366

natural cures of . . v 233

nature of . . . v 230, xiii 447

nitre in . . . . xii 247

opium in . . vi 369, ix 274

pericarditis from . ix 485, xxiv 567

purgatives in . . . vi 369

sudorifics in . . . vi 368

suppuration in . . vi 366

termination of . . vi 366

treatment of iii 555, vi 370, xiv 415, xx 184-8, xxi 108, xxiv 45

with pneumonia . . xx 184

a disease sui generis . . vi 379

ages most liable to . . vi 359

and chorea, connexion of . ix 433

dysmenorrhœa, connexion of xviii 522

gout, diagnosis of . . xiv 415

Dr. Todd on . . xvi 460

on the identity of . vi 376

pathology of . . xii 120

neuralgia, diagnosis of . xiv 412

pericarditis, relation of . xx 183

syphilis, diagnosis of . vi 372

aponeuroses the seat of . . iii 78

articular . . . . . vi 358

invasion of . . . vi 360

best climates for . . . xii 164

bleeding in, causing heart-affections xiii 452

Bouillaud's bleeding in . . vi 367

discovery in . . . vi 364

brain-affections in . . . xiii 456

Brera's pathology of . . i 566

calomel and opium in . . iii 555

capsular, deposits in . . xiii 458

distinction of . . . xiii 458

duration of . . . xiii 460

in the sexes . . . xiii 460

metastasis in . . . xiii 459

peculiarities of . . xiii 457

suppuration in . . xiii 458

treatment of . . . xiii 460

carburet of sulphur in . . ii 252

caused by cold and moisture . vi 359

causes and prevention of . vi 17

characteristics of . . . vi 353

chronic, diagnosis of . . vi 371

duration of . . . vi 371

prognosis in . . . vi 371

treatment of . . . xiv 417

classed with the neuroses . i 254

cod-liver oil in . . . xiv 419

cold and heat in . . . xiv 420

cold bathing in . . . ii 532

constitutional origin of . . xvi 468

cured by warm and cold water i 499

cutaneous deposit in . . xiii 459

diet and regimen in . . xiv 421

Rheumatism, different forms of . vi 353

diseases predisposing to . . xiv 415

diuretic mixture for . . xiv 417

Dr. Bene on . . . . i 7

Dr. Hope on treatment of . iii 555

Dr. Macleod on . . . xiii 445

Dr. Todd on capsular . . xvi 470

effect of climate on . . xiii 423

season on . . . vi 371

exacerbation of, at night . xiii 447

exciting cause of . . . xiii 446

external remedies for . . xiv 420

first stage, treatment of . . xiv 416

formula of gold for . . xiv 418

from copaiba . . . . viii 280

lead, described . . x 448

suppressed catamenia . xviii 522

leucorrhœa . xviii 522

heat in . . . . . xix 186

hectic fever in . . . vi 371

hereditary nature of . . vi 359

hot-air bath for . . . xvii 485

hydropathic treatment of . xvi 471

hypertrophy of heart after . xiii 457

Indian hemp in . . . x 226

inflammatory or not . . xvi 468

in American army . . . xiii 433

in India, Mr. Malcomson on . v 130

sequelæ of . . v 131

in Indian army . . . xxiii 126

causes of . . xxiii 127

in relation to the uterus . xvi 470

insolation in . . . . xiv 420

its disposition to recur . . vi 359

its treatment by homœopathy . xxii 577

joints most liable to . . xiii 447

lead, diagnosis of . . . x 448

metals and earths in . . xiv 418

metastasis in . . . vi 361, ix 486

M. Piorry on . . . vi 147

medicines for the blood in . xiv 418

metastatic, a cause of . . viii 325

metastasis of, to the brain . xxii 427

to the head . . . xiv 413

mild chronic articular . . vi 371

mineral hot baths in . . xiv 336

morbid anatomy of . . vi 372

most frequent in males . . vi 359

moxa in . . . . . xiv 581

muscular . . . . vi 354

of head . . . . vi 354

of the limbs . . . vi 358

variety of . . . xiii 460

nature and cure of . . vi 352

of the pain in . . vi 361

nervines for . . . . xiv 418

night exacerbations in . . vi 371

of natives of India . . . v 130

the abdominal parietes . vi 356

of alimentary canal . . . vi 374

bladder . . . . vi 376

diaphragm . . . . vi 373

dura mater . . . xiv 413

Rheumatism of Europeans in India . v 130

of heart . . . . . vi 362, 374

caution on . . . vi 364

diagnosis of . . . vi 374

Dr. Watson on . . vi 365

signs of . . . . vi 363

stethoscopic signs of . xiii 454

neck . . . . . . vi 355

palpebræ, case of . . vi 355

periosteum . . . vi 376

peritoneum . . . xiii 460

skin . . . . . . xiii 234

spinal cord . . . xiv 412

sclerotic . . . . vi 376

teeth . . . . . vi 376

testicle . . . . xiii 447

thoracic muscles . . . vi 355

uterus . . . . xiii 460

pathology of . . . . . i 254

pleurisy in . . . . . xiii 459

premonitory, powder for . . xiv 416

treatment of . . . xiv 415

prevalent in warm climates . xiii 432

preventive of relapse of . . vi 359

puerperal state liable to . . xviii 522

pulse in . . . . . ix 485

relative prevalence of . . vi 14-16

remarks on the cause of . . xvii 484

remedies for . . . . . xvii 485

seat of . . . . vi 353, xiv 414

second stage, treatment of . xiv 416

spinal diseases from . . iv 379

spinal, in dogs . . . xxiv 89

state of the blood in . . xiv 414

the fever in . . . . . vi 360-1

the nervous system in . . xiv 414

the paroxysms in . . . vi 361

third stage, treatment of . . xiv 417

tophaceous secretions in . . vi 371, 373

transmission of, by suckling (?) xx 215

treated by the ballota lanata . i 567

treatment of . . . xvi 471, xx 268

by nitre . . . xxi 372

treatments of, compared . . xiii 450

universal, treatment of . . xiv 417

various narcotics in . . xiv 417

visceral, M. Chomel on . . vi 373

with endocarditis . . . vi 363

pericarditis . . . vi 362

Rheumatismal renal inflammation . x 303

Rhinoplastic, etymology of . . vii 400

Rhinoplastic operation, by Dr. Warren v 245

Dieffenbach's . xxi 294

from the arm . xxi 302

Indian . . . vii 392

remark on . . vii 391

operations, cases of . . xix 399

partial . . . . . xxi 296

Rhinoplasty . . . . . . vii 400

Rhizados, implantation of teeth of . xx 394

Rhonchi, catarrhal . . . . iv 302

classification of the various . ix 318

in phthisis . . . . . ix 329

Rhonchi, laws of coexistence of . ix 318

vesicular, in pneumonia . . ix 317

Rhonchus, peculiar vesicular . . ix 314

remarks on the crepitant . . iv 298

Rhubarb, in venereal ulcers . . xiv 576

nephritic attack from use of . xvii 25

oxalic acid contained in . . xvii 25

Rhythm, morbid symptoms with . xxiv 318

Rhythmical disease, and recurrent . xxiv 317

Riadore, Dr., his contemptible work xiv 532

on oxygen, or vital air . . xix 232

on spinal irritation . . . xiv 532

Ribaut, M., his hint on vaccination vi 490

Rib, on motion of the first . . xx 135

removal of a whole . . . xv 245

Ribs, articulations of, in the sexes . xvii 254

case of enlargement of . . iii 78

congenital luxation of . . xxi 401

fracture of . . . . . vii 554

by contre-coup . . v 574

Lisfranc on . . xiv 11

inflammatory enlargement of . iii 77

operations for excision of . vi 548

spontaneous fracture of . . vi 227

Rice-diet, remarks on . . . xvii 30

Rice, nutritious elements in . . xvii 19

Riches, their influence on longevity . i 195

Richet, M., on Pott's disease . . i 574

on white swellings . . . xxi 124

Richter, Dr., on diseases of the bones xii 154-9

his medical contributions . xv 405, 410

on infantile gangrene . . vii 470

on nux vomica in dysentery . i 256

Rickets, effect of civilization on . xviii 247

effects of, on the skull . . xviii 371

erratic character of . . xvii 377

in children, report on . xvii 566, xx 564

infantile life not free from . xvii 376

limited to the cranium . . xvii 377

morbid anatomy of . . . xii 187

nature and treatment of . . xii 158

Ricord, M., his new urethroplasty xii 543

on blenorrhagia in women . i 271

on venereal diseases . . x 381, 417

Ridge, Dr., his Glossology . . xvii 524

strictures on . xvii 524

Riecke, Dr., on burial-grounds, &c. xv 513

on hernia litrica . . . xiv 360

Rieken, Dr., on ammonia in scarlatina xvi 512

Riga, medical archives of . . xii 430

Rigby, Dr., his edition of Dr. W. Hunter xvii 530

his memoranda in midwifery . iii 494

his midwifery . . . xii 461

on dysmenorrhœa . . . xviii 522

Rigg, Mr., on the growth of plants . vi 552

Right and wrong, on a standard of xxiv 231, 241

Rings, inguinal and femoral, action of xx 139

Rigidity, of limbs, a fatal sign in typhoid fever . . . ii 37

after asphyxia, Orfila on . ii 425

Rigor mortis . . . . . . xix 259

Bruecker on . . . xvii 252

Rigors in puerperal disease . . xiii 106

